# Targeted degradation of sICOSL reverses cytotoxic T cells dysfunction

**DOI:** 10.1186/s40164-025-00692-x

**Published:** 2025-07-24

**Authors:** Zhenghao Wu, Peng Zheng, Ruobing Qi, Yunxiao Xiao, Zihan Xi, Lei Dai, Tao Chen, Qianheng Wang, Furong Zhang, Rong Wang, Zimei Tang, Xiangwang Zhao, Jie Tan, Jie Ming, Ping Lei, Chunping Liu, Tao Huang

**Affiliations:** 1 Department of Breast and Thyroid Surgery, Union Hospital, Tongji Medical College, Huazhong University of Science and Technology, Wuhan, China; 2 Department of Immunology, School of Basic Medicine, Tongji Medical College, Huazhong University of Science and Technology, Wuhan, China; 3 Guangdong Provincial Key Laboratory of Malignant Tumor Epigenetics and Gene Regulation, Medical Research Center, Sun Yat-Sen Memorial Hospital Sun Yat-Sen University, Guangzhou, China; 4 Department of Oncology, Tongji Hospital, Tongji Medical College, Huazhong University of Science and Technology, Wuhan, China; 5 Division of Cardiology, Department of Internal Medicine, Tongji Hospital, Tongji Medical College, Huazhong University of Science and Technology, Wuhan, China

**Keywords:** Breast cancer, Cytotoxic T cells, Immune evasion, Protein targeted degradation, ICOSL

## Abstract

Dysfunction of cytotoxic T cells (CTL) remains a major cause of tumor immune evasion and is correlated with poor cancer survival. Here, we found that increased soluble form of ICOSL (sICOSL) induced CTL dysfunction and was associated with shorter survival of patients with breast cancer. sICOSL emerged as a formidable adversary to CTLs, by directly triggering ICOS internalization and subsequent degradation—a critical blow to the co-stimulatory machinery essential for CTL activation. Our research shows that dipeptidyl peptidase-4 (DPP4) mainly breaks down sICOSL. Notably, certain chemotherapeutic drugs activate the histone methyltransferase Enhancer of zeste homolog 2 (EZH2), which in turn suppresses DPP4 expression. To address this issue, we have developed nanobody-DPP4 fusion proteins that can specifically degrade sICOSL, achieving substrate selectivity and tumor targeting. Overall, This work unveils that sICOSL orchestrates CTL dysfunction, and establishs targeted degradation of sICOSL as a new strategy for immunotherapy.

**Supplementary Information:**

The online version contains supplementary material available at 10.1186/s40164-025-00692-x.

## Introduction

Breast cancer is the most common malignant tumor globally, and it ranks second among cancer-related causes of death in women [1]. Chemotherapy is one of the primary treatment modalities for breast cancer. However, the complete pathological response rate after neoadjuvant chemotherapy is only 18–48% [2]. Therefore, identifying non-responders and sensitizing them to chemotherapy remain challenging issues in the clinical practice [3].

Traditionally, chemotherapeutic agents were believed to exert their anti-tumor effects primarily through direct tumor cell killing. However, mounting evidence suggests that the tumor immune microenvironment (TME) also plays a significant role [4]. On the one hand, a “hot” TME enhances the effectiveness of chemotherapy. Breast cancer patients with high levels of intratumoral lymphocyte infiltration exhibit longer overall survival after chemotherapy [5]. Conversely, cytotoxic T cells (CTLs) depletion from tumor tissue significantly weakens chemotherapy efficacy [6]. On the other hand, chemotherapy drugs can directly transform the TME from “cold” to “hot”, by inducing immunogenic substances release, thereby activating CTLs [7]. However, research indicates that these CTLs become dysfunctional after chemotherapy, which limits their ability to kill tumor cells and weakens the overall anti-tumor effect [8]. Therefore, a thorough understanding of the mechanisms underlying CTL dysfunction is crucial for improving the effectiveness of chemotherapy in breast cancer treatment.

Extracellular soluble proteins are a crucial component of the TME, including cytokines, chemokines, and hormones. These soluble proteins play a vital role in regulating the localization, distribution, and function of immune cells [9, 10]. Research indicates that the stress induced by chemotherapy leads to the release of various soluble proteins from tumor cells [8, 11]. It is speculated that some chemotherapy-induced factors impact the anti-tumor activity of CTL, but the attribute and source of these factors are not clear.

However, accurately quantifying the content of soluble factors within tumor tissues poses a challenge. Traditional methods (such as immunohistochemistry, western blot, and mass spectrometry) struggle to distinguish among intracellular, membranous and extracellular proteins [12]. Building on the literature, we had previously established methods to extract tumor interstitial fluid and identify tumor-derived extracellular factors with high sensitivity and specificity [13, 14]. In this study, we combined this approach with liquid-phase multiplex protein quantification technology, enabling high-throughput screening of soluble proteins. Here, we identified soluble ICOSL (sICOSL) as a chemotherapy-induced soluble factor that mediates CTL dysfunction. Through multiomics analyses of clinical samples and biological experiments in cell and mouse models, we discovered a previously uncharacterized EZH2-DPP4-sICOSL-ICOS pathway that regulates cancer-CTL communication, tumor immune evasion, and the breakdown of chemoimmunotherapy. Notably, we developed a targeted protein degradation (TPD) therapy to reverse the immune evasion mediated by sICOSL.

## Results

### sICOSL indicates immune evasion of breast cancer

**Fig. 1:**
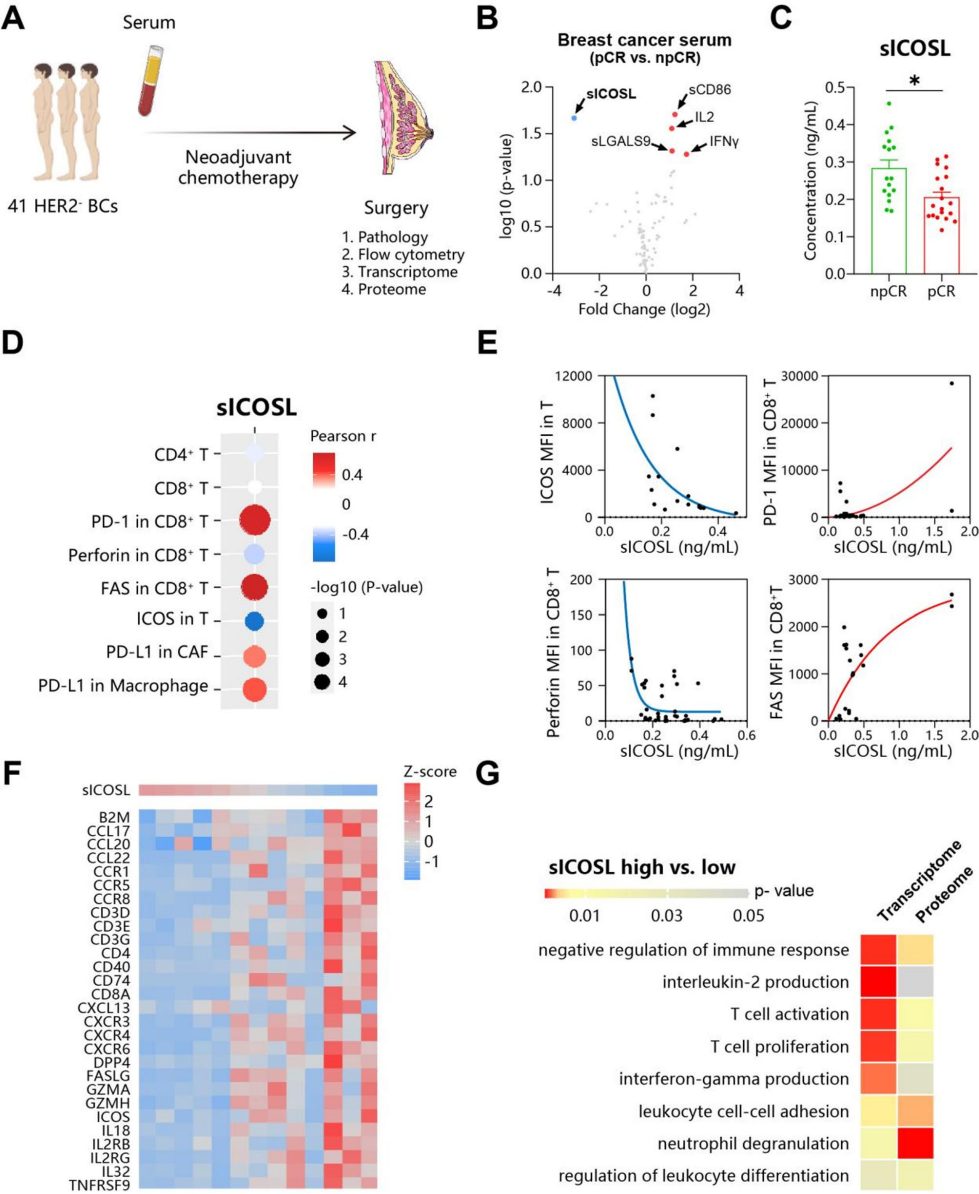
sICOSL indicates immune evasion of breast cancer. (**A**) Diagram representation of HER2 negative breast cancer patients cohort, who were treated with neoadjuvant chemotherapy and surgery. The serum during chemotherapy and surgery speciman after chemotherapy were collected. (**B**) Volcano plot showing soluble proteins changed in patients’ serum who are pathology complete response (pCR) compare with non-pCR (npCR) after chemotherapy, as determined by bead-based immunoassay or ELISAs. For each soluble protein, average concentration during chemotherapy are upregulated (red; p < 0.05, FC > 2) or downregulated (blue; p < 0.05, FC<-2). (**C**) sICOSL concerntration in patients’ serum with different response to chemotherapy. (**D**-**G**) The relationship between sICOSL level and tumor microenvironment. (**D**-**E**) Flow cytometry analysis revealed several parameters in the post-chemotherapy tumor, including percentage of CD4 + and CD8 + T cells in immune cells, median fluorescence intensity (MFI) of several markers in T cells, CAF and macropahge. The correlation coefficient (**D**) and representative non-linear regression diagram (**E**) are shown. (**F**) Heatmaps of the RNA sequencing data of post-chemotherapy tumor. Z-score of sICOSL concerntration and representative genes expression are shown. (**G**) Immune-related genesets are enriched in tumors from patients with high sICOSL level. Gene set enrichment analysis are performed based on transcriptome and proteome data

To screen for potential factors, we prospectively enrolled 35 women with early and locally advanced HER2-negative breast cancer undergoing neoadjuvant chemotherapy (Fig. 1A, the cohort characteristics are summarized in Supplementary Table 1). Pre-treatment serum and post-treatment surgical samples were collected. Immune-related regulatory proteins were detected in the serum based on LEGENDplex multiplex assays. Tumor samples were profiled by multicolor flow cytometry, RNA sequencing and Tandem Mass Tag 16plex (TMT16)-based proteome analysis. Chemotherapy (including taxane, anthracycline, platinum-based regimen) was administered for a median of 18 weeks. Response was assessed at surgery using the residual cancer burden (RCB) classification. On completion of neoadjuvant treatment, 19 (54%) had a pathological complete response (pCR), 1 (3%) had a good response (RCB-I), 6 (17%) had a moderate response (RCB-II) and 9 (26%) had extensive residual disease (RCB-III). We compared serum protein abundance between patients with pCR and non-pCR (including RCB-I/II/III) and identified soluble ICOSL (sICOSL) as an adverse prognosis marker (Fig. 1B, C). Notably, among patients with poor response to neoadjuvant therapy (non-pCR), sICOSL levels showed significant individual variation. Approximately half (7/16) of these patients had sICOSL concentrations exceeding 0.31 ng/mL, the maximum level observed in all pCR patients. This suggested that in this group of patients, sICOSL was associated with worse clinical outcomes.

Furthermore, we analyzed the relationship between sICOSL concentration and immune cell phenotype measured through flow cytometry. It was found that sICOSL was associated with more PD-1 and FAS expression and less perforin expression in CD8^+^ T cells (Fig. 1D, E). Notably, only the function but not number of T cell infiltrates was affected by sICOSL. Transcriptome data revealed that sICOSL was negatively correlated with expression of cytokines, chemokines, T cell surface and cytotoxic markers (Fig. 1F). Notably, GSEA analysis utilizing transcriptome and proteome data also linked sICOSL levels to impaired T cell function, including activation, proliferation, and the production of IL-2 and IFN-γ (Fig. 1G). In summary, high sICOSL levels indicated dysfunction of tumor-infiltrating T cells, suggesting immune evasion in the post-chemotherapy TME.

### sICOSL promotes tumor growth in immunocompetent hosts

**Fig. 2:**
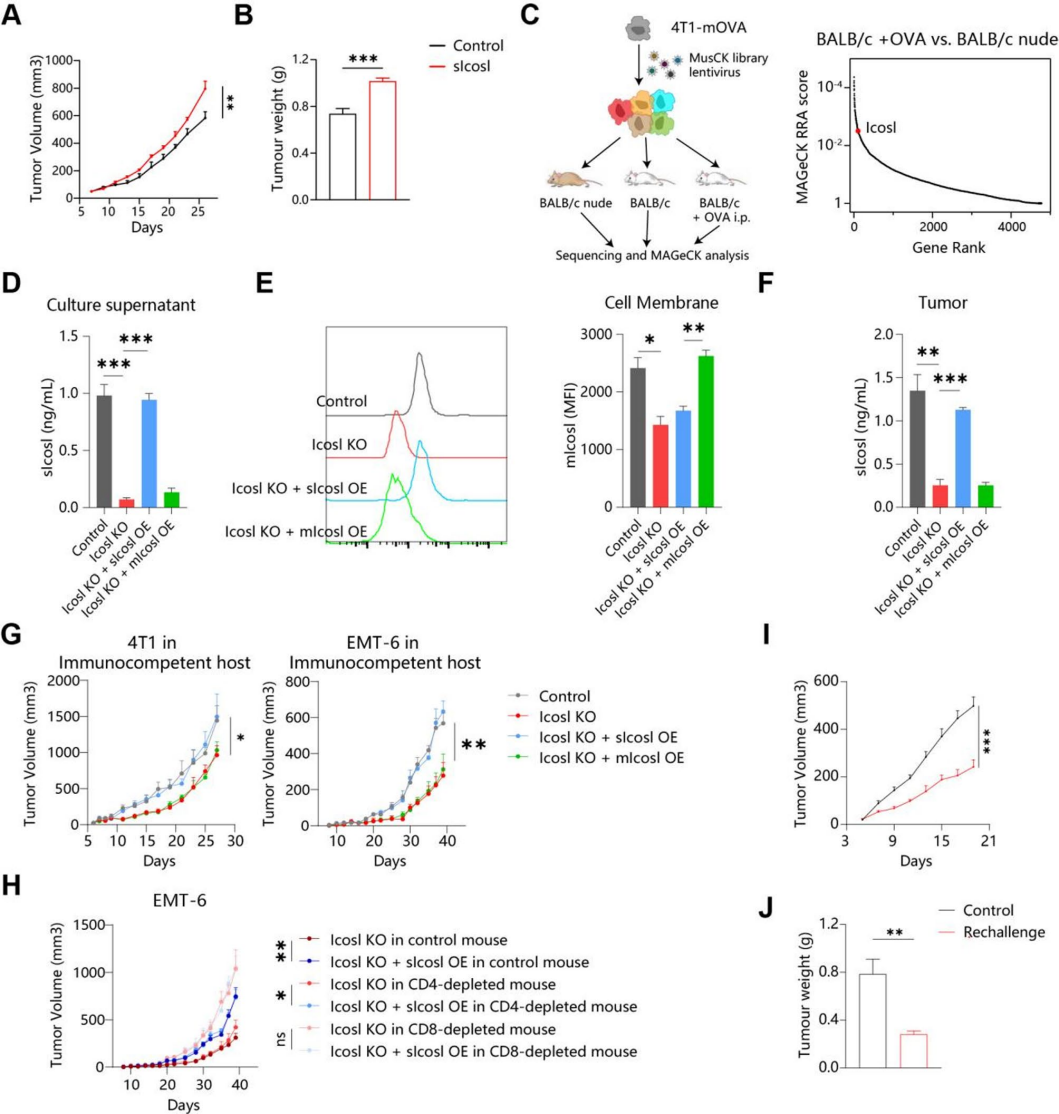
sICOSL promotes tumor growth in immunocompetent hosts. (**A**-**B**) Wild-type 4T1 cells were injected into the inguinal mammary gland. Once tumors were palpable, mice were injected intratumorally (i.t.) with PBS or sIcosl (1 mg/kg) every two days. Tumor volume (**A**) and weight (**B**) from mice were shown (n = 8). (**C**) Workflow of the MusCK in vivo screens reported by Wang XQ et al. The top depleted genes in immunocompetent versus immunodeficient (nude) hosts in the MusCK screens were shown (right). (**D**-**E**) sIcosl levels in the culture supernatant (**D**) and membrane-associated mIcosl abundance (**E**) of 4T1 cells expressing different Icosl in vitro. (**F**) sIcosl concentration in the tumor interstitial fluid of 4T1 tumors expressing different Icosl in vivo. (**G**) Tumor growth of 4T1 and EMT-6 cells expressing different Icosl in immunocompetent mice (n = 8). (**H**) Tumor growth of EMT-6 cells expressing sIcosl in control or CD4/CD8-depleted mice. (**I**-**K**) First challenge with PBS or 4T1 Icosl-KO cells on one side of the inguinal mammary gland (1st), and second challenge after 28 days with 4T1 Icosl-WT tumor cells on both sides of the mammary gland (2nd). Tumor volume (**I**) and weight (**J**) from rechallenged mice were shown (n = 8). KO: Knock out; OE: Over expression

sICOSL was associated with chemotherapy failure and T cells anergy, suggesing sICOSL might directly promote cancer progression. We intratumorally injected murine sIcosl into mouse breast cancer, and observed that sIcosl directly promoted tumor growth (Fig. 2A-B). Then, we tried to explore whether sICOSL depletion might also hinder tumor progression. Wang XQ et al. transduced the murine lentiviral CRISPR (Clustered Regularly Interspaced Short Palindromic Repeats)-Cas9 knockout (MusCK) library into membrane-bound ovalbumin (mOva)-expressing 4T1 cells and implanted infected cells into the mammary fat pads of BALB/c nude hosts, immune competent BALB/c hosts, and BALB/c hosts vaccinated with OVA prior to transplantation (Fig. 2C) [15]. sgRNAs targeting Icosl were depleted in tumors engrafted in immunocompetent hosts compared with immunodeficient mice (Fig. 2C). Thus, it is possible that increased sIcosl release in tumors has an immunosuppressive function, which would explain why knockout (KO) of Icosl genes enhances immune-mediated killing of breast cancer. To confirm our findings, we deleted Icosl in two mouse breast cancer cells (4T1 and EMT-6) (Figure S1A). However, the knockout of Icosl simultaneously downregulated both the membrane-bound Icosl (mIcosl) and the soluble Icosl (sIcosl). To differentiate between these two forms, we selectively overexpressed sIcosl (lacking the transmembrane domain and equipped with a signal peptide) and mIcosl (with mutations in the shedding site) in the Icosl-KO breast cancer cell lines. Both in vitro and in vivo assays confirmed that these modifications altered the level distribution of different forms of Icosl in breast cancer (Fig. 2D-F). Upon transplanting these cell lines into immunocompetent hosts, it was observed that the knockout of Icosl significantly suppressed tumor growth, while the re-expression of sIcosl, but not mIcosl, reversed this effect (Fig. 2G). Thus, sIcosl is one of the factors mediating the progression of breast cancer. However, it remains to be determined whether Icosl expressed by cancer cells directly affects the cancer cell phenotype or acts on the immune microenvironment. In vitro experiments showed that Icosl does not influence the proliferation, migration, and invasion of cancer cells (Figure S1A-D). In addition, Icosl also had no effect on tumor growth and survival in Rag1^−/−^ mice lacking T and B cells (Figure S1E-F). To further pinpoint the specific immune cell subsets affected by sIcosl, we first depleted the corresponding T cell subsets in mice using anti-CD4 or anti-CD8 monoclonal antibodies. Subsequently, we transplanted cancer cells overexpressing sIcosl into the mice’s mammary fat pads and compared the resulting tumor growth curves. We found that in mice depleted of CD8 + T cells, the overexpression of sIcosl no longer promoted tumor growth, whereas the effect persisted in mice depleted of CD4 + T cells (Fig. 2H). This indicates that sIcosl primarily mediates the evasion of cancer cells from immune surveillance by CD8 + T cells. On the other hand, wild type (WT) tumor cells injected into immunocompetent mice that were previously challenged with sIcosl-deficient tumor cells also grew slowly (Fig. 2I-J), suggesting that sIcosl-defecient tumor cells can vaccinate hosts against wild type tumors. In summary, breast cancer potentially release sIcosl to promote immune evasion.

### Tumor-released sICOSL mediates T cell dysfunction

**Fig. 3:**
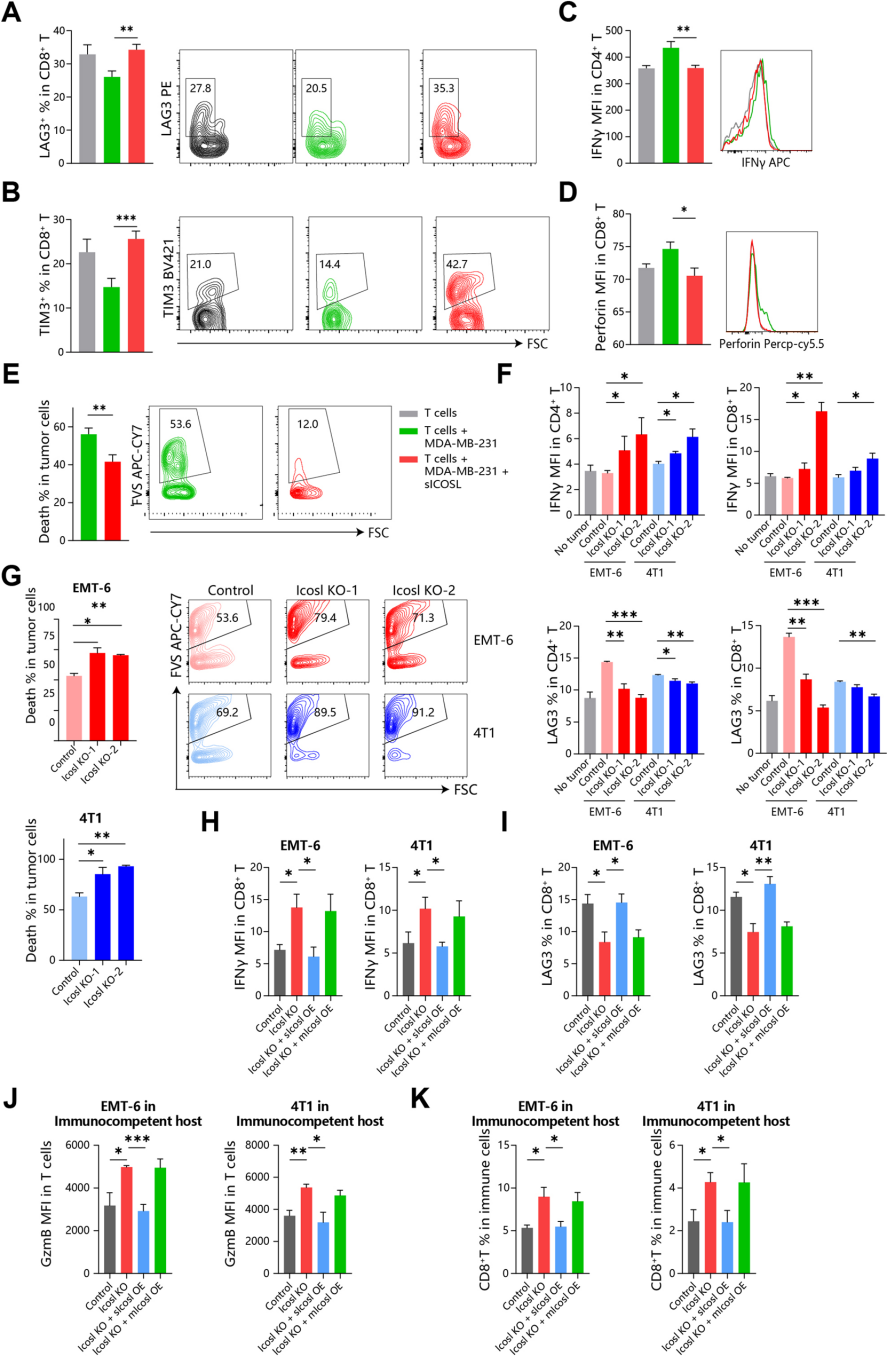
Tumor-released sICOSL mediates T cell dysfunction. (**A**-**E**) Tumor-antigen activated T cells were co-cultured with CellTrace-labeled MDA-MB-231 cells and treated with sICOSL for 18 h. T cell exhaustion was evaluated through the expression of LAG3 (**A**) and TIM3 (**B**) in CD8 + T cells. Activation markers, including IFNγ (**C**) and perforin (**D**), were stained in CD4 + and CD8 + T cells, and dead tumor cells were defined as PI + CellTrace + cells using flow cytometry (**E**). (**F**-**G**) Icosl-KO/WT 4T1 and EMT-6 cells were labeled with CellTrace and co-cultured with tumor-antigen activated T cells. Expression of IFNγ and LAG3 in CD4 + and CD8 + T cells (**F**) and dead tumor cells (**G**) was assessed. (**H**-**I**) 4T1 and EMT-6 cells expressing different Icosl were labeled with Cell Trace and co-cultured with tumor-antigen activated T cells. Expression of IFNγ (**H**) and LAG3 (I) in CD8 + T cells were evaluated. (**J**-**K**) Tumors from 4T1 and EMT-6 cells expressing different Icosl in immunocompetent mice were dissected and analyzed using flow cytometry. Granzyme B (GzmB) expression in T cells (**J**) and T cell infiltration (**K**) were shown

ICOS, the receptor for ICOSL, is mainly expressed on activated cytotoxic T cells, helper T cells, and regulatory T cells [16]. Therefore, we hypothesized that sICOSL released by cancers may modulate T cell function to facilitate immune evasion. First, we evaluated the direct effect of sICOSL on T cells in vitro and treated tumor-antigen activated T cells with sICOSL. sICOSL enhanced the LAG3 and TIM3 expression in CD8^+^ T cells, and respectively downregulate IFNγ and perforin in CD4^+^ T cells and CD8^+^ T cells (Fig. 3A-D). Therefore, sICOSL directly promoted T cells exhaustion and inhibited the cytotoxicity of T cells. Consistently, dead tumor cells were also reduced after sICOSL treatment (Fig. 3E). However, it remained unclear whether tumor-released sICOSL has the same effect on T cells as exogenous sICOSL. So we compared the T cells function in Icosl-KO and WT tumor in vitro and in vivo. As Icosl depleted, breast cancer didn’t release sIcosl and also significantly enhanced IFNγ expression and inhibited LAG3 presence in both CD4^+^ and CD8^+^ T cells (Fig. 3F). Notably, T cells cocultured with Icosl-KO cell lines had the same LAG3 levels with control T cells (Fig. 3F). Icosl-KO breast cancer completely aborted the ability to induce T cell exhaustion (Fig. 3F), and also enhanced T cells cytotoxicity (Fig. 3G). Therefore, tumor-released ICOSL mediates T cell dysfunction. To further distinguish the effects of ICOSL with different localizations, we compared the impact of separately overexpressing sIcosl and mIcosl in Icosl-KO breast cancer cells on T cell phenotypes (Fig. 2D-F). Accordingly, only the overexpression (OE) of sIcosl significantly induced CD8 + T cell exhaustion and diminished their antitumor activity, while mIcosl did not markedly affect CD8 + T cells (Fig. 3H-I). Similar results were also revealed in sIcosl-OE/mIcosl-OE/Icosl-KO/WT 4T1 and EMT-6 tumor in the immunocompetent mice mammmary. T cells from Icosl-KO tumors exhibited heightened cytotoxicity, characterized by increased granzyme B expression, whereas overexpression of sIcosl abrogated this effect (Fig. 3J). Notably, a greater enrichment of CD8 + T cells was observed in Icosl-KO tumors compared to sIcosl-OE tumors, suggesting that sICOSL could also hinder T cell infiltration (Fig. 3K). In conclusion, tumor-derived sICOSL but not mICOSL facilitated T cell dysfunction by inhibiting T cell infiltration and turning cytotoxic T cells exhausted.

The mechanism about how sICOSL promoted T cell dysfunction remains to be explored. ICOS, the classical receptor for ICOSL, deliver a positive co-stimulatory signal to T cells [17]. In addition, sICOSL was also reported to regulate ICOS expression in the membrane surface of T cells [18]. Therefore, we speculated that sICOSL could induce ICOS internalization to impede the activation of antitumor cytotoxic T cells. In vitro experiments revealed that sICOSL directly downregulate surface expression of ICOS (Figure S2A, B). Consistently, Icosl-KO breast cancer cell lines directly induce ICOS expression in CD4^+^ and CD8^+^ T cells (Figure S2C). The similar phenomenon was also found in the Icosl-KO/WT mammmary tumor of immunocompetent mice. Increased ICOS-positive CD4^+^ and CD8^+^ T cells enriched in Icosl-KO tumor (Figure S2D, E). However, the number of ICOS^+^ Treg wasn’t influenced by sIcosl depletion, possibily because ICOS was constitutively highly expressed in Treg and insensitive to exogenous factors [16]. In addition, ICOS was also detected to express differentially in other lymphocytes. The allograft breast cancer models revealed that only ICOS expression in T cell dynamically changed during tumor progression, and thus was possibly affected by other factors in the TME (Figure S2F, G). After rapid escalation in the first two weeks, T cell-expressed ICOS gradually reduced (Figure S2G). However, the concentration of sICOSL continued to increase throughout the entire course, while the levels of mICOSL remained stable (Figure S2H). So there is hysteresis in sICOSL-dependent ICOS downregulation. In addition, we performed the immunofluorescence and confirmed that ICOS was endocytosed and trafficked to the lysosome after sICOSL induction (Figure S2I). In summary, tumor-released sICOSL induce the endocytosis and degradation of ICOS to cause T cells dysfunction.

### Chemotherapy downregulate DPP4 to inhibit the degradation of sICOSL

**Fig. 4:**
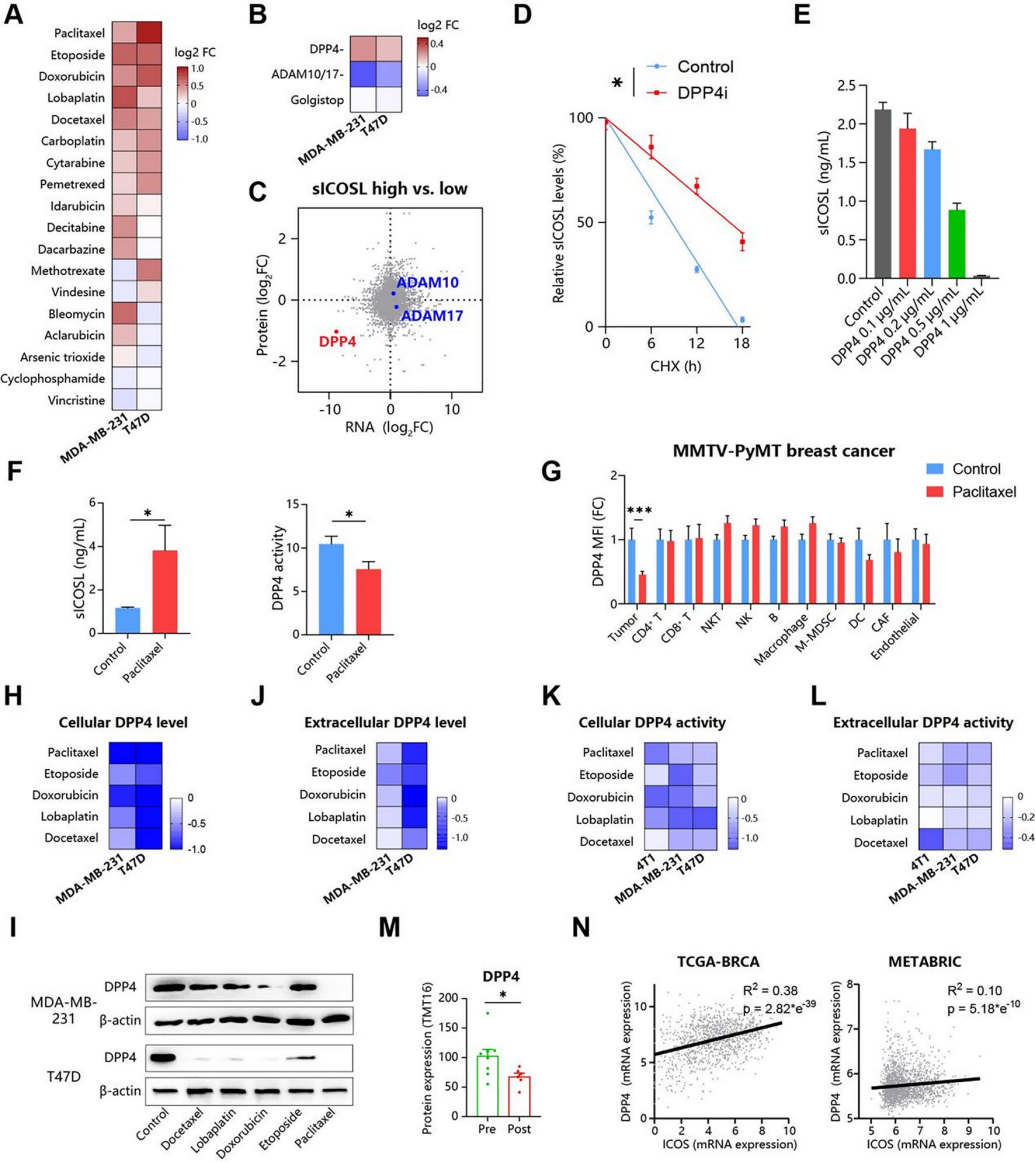
Chemotherapy downregulate DPP4 to inhibit the degradation of sICOSL. (**A**) Heatmaps illustrating the Log2 fold change (values greater than 0 indicate an increase) in sICOSL concentration within the culture supernatant of cell lines exposed to various chemotherapeutic drugs, in comparison to untreated controls. (**B**) sICOSL release of cell lines treated with inhibitors of DPP4 (100µM), ADAM10/17 (1mM) and protein transport (1x). (**C**) Volcano plot showing genes expression difference in breast cancer tissues with high and low sICOSL levels, as determined by transcriptome and proteome. Representative genes were shown (red for significantly differentially expressed genes in both dimensions). (**D**) The effect of DPP4 inhibitors on sICOSL degradation was analyzed using a CHX chase assay. (**E**) The cancer cell supernatant was incubated with varying concentrations of DPP4 for 12 h, and the sICOSL concentration was measured. (**F**, **G**) MMTV-PyMT transgenic mice received paclitaxel (i.p., q3d). The primary mammary tumors were dissected and analyzed with flow cytometry. (**F**) sICOSL concentration and DPP4 activity in the tumor interstitial fluid. (**G**) DPP4 expression in chemtherapy group were normalized of based on control group for each cell cluster. (**H**-**L**) The activity and expression level of DPP4 in cell lines treated with five chemotherapeutic drugs. Cellular (**H**, **I**) and extracellular (**J**) DPP4 level were individually measured using WB and ELISA. Cellular (K) and extracellur (**L**) DPP4 activity were detected through GP-pNA based assay. (**M**) DPP4 expression in pre- and post- chemotherapy breast cancer samples according to TMT16-based preotome. (**N**) The correlation between DPP4 and ICOS expression in TCGA-BRCA and METABRIC datasets

Aforementioned results confirmed tumor-released sICOSL induced T cells dysfunction and indicated poor prognosis during chemotherapy of breast cancer patients. It remained to be explored how chemotherapy influenced sICOSL production. We screened common chemotherapeutic drugs to evaluate their ability to change the sICOSL concentration in the culture supernatant of breast cancer cell lines (Fig. 4A). Classical cytotoxic drugs for breast cancer, such as taxoids (paclitaxel, docetaxel), platinum (lobaplatin, carboplatin) and anthracyclines (doxorubicin), significantly increase sICOSL secretion with Log2 Fold Change (FC) > 0.5(Fig. 4A). Other drugs had little effect on sICOSL release. Thus, it raised the questions about how these cytotoxic drugs induced sICOSL secretion. Therefore, we reanalyzed the transcriptome and proteome data of the breast cancer cohort that received at least one of the sICOSL-inducing drugs, including taxoids, platinum, and anthracyclines (Fig. 1A). It was reported that ADAM10 and ADAM17 could shed ICOSL from plasma membranes [18]. The levels of sICOSL were significantly suppressed by ADAM10/17 inhibitors and were unaffected by Golgistop, confirming that sICOSL was released through ADAM-dependent shedding of mICOSL, not via Golgi-derived secretory vesicles (Fig. 4B). However, using the transcriptomic and proteomic datasets from the above-mentioned breast cancer patient cohort, it was found that the expression levels of ADAM10/17 were comparable in breast cancers with low and high sICOSL levels (Fig. 4C), suggesting that ADAM10/17 is not a key regulator of sICOSL levels in breast cancer. Notably, another protease, dipeptidyl peptidase 4 (DPP4), was significantly enriched in breast cancers with low sICOSL levels (Fig. 4C). DPP4 inhibitors markedly increased the levels of sICOSL in the culture supernatant (Fig. 4B). Cycloheximide (CHX) chase assays revealed that DPP4 inhibitors extended the half-life of sICOSL from 8.7 h to approximately 16.3 h (Fig. 4D). Therefore, DPP4 emerged as the major key gene influencing sICOSL levels in the tumor microenvironment following chemotherapy. DPP4, a serine protease known to cleave and inactivate various proteins, suggests its potential involvement in the degradation of sICOSL. To substantiate this, recombinant DPP4 was introduced to the supernatant of breast cancer cells, and ELISA analysis demonstrated that the concentration of sICOSL decreased progressively with increasing DPP4 concentrations (Fig. 4E). This indicates that DPP4 is directly involved in the degradation of sICOSL. DPP4 may also reduce the source of sICOSL by degrading mICOSL. Further analysis revealed that when recombinant DPP4 was co-incubated directly with breast cancer cells and subsequently analyzed by flow cytometry using an anti-ICOSL antibody, the level of mICOSL remained unaffected by the concentration of DPP4 (Figure S3A). Consistently, DPP4 inhibitors did not enhance mICOSL expression (Figure S3B). In summary, DPP4 specifically mediated the degradation of sICOSL rather than mICOSL in the breast cancer microenvironment.

The regulation and source of DPP4 in the breast cancer microenvironment after chemotherapy were still unclear. We injected transgenic MMTV-PyMT (mouse mammary tumor virus-polyoma middle tumor-antigen) mouse mammary gland carcinoma and lung metastasis model with paclitaxel. Interstitial fluid from tumor was enriched with sICOSL and had lower DPP4 activity (Fig. 4F). Similar results were also found in the interstitial fluid of metastatic lung (Figure S3C). Therefore, chemotherapeutic drugs such as paclitaxel could downregulate DPP4 in the TME. CD26/DPP4 is widely expressed on endothelial and epithelial cells of various solid organs as well as in most immune cells [19]. To identify which cells are affected by paclitaxel, DPP4 expression in different cell clusters was detected through flow cytometry (Fig. 4G, S3D). Notably, paclitaxel decreased DPP4 expression only in tumor cells of primary breast cancer (Fig. 4G). As for microenvironment of lung metastases, paclitaxel also downregulated DPP4 in tumor cells, NK cells, macrophage and DCs, indicating more complicated effect of paclitaxel in the lung (Figure S3E). Among all cell clusters from both carcinomas in situ and lung metastases, tumor cells were most sensitive to paclitaxel, with DPP4 expression being reduced by more than half (Fig. 4F, S3F). In addition, the DPP4 activity continuously reduced along with the rise of sICOSL concentration during the whole course of tumor progression (Figure S2C, S3G). Therefore, we speculated that paclitaxel mainly inhibited DPP4 expression in the tumor cells to upregulate the sICOSL levels.

To confirm the effect of chemotherapy on DPP4 expression, we selected several drugs with high sICOSL-releasing levels, including paclitaxel, etoposide, doxorubicin, lobaplatin, and docetaxel (Fig. 4B). DPP4 is expressed as a transmembrane protein and is also released into body fluids as a soluble form [19]. So we individually detected cellular and extracellular DPP4 levels through western blots and ELISA, and found these drugs reduced both DPP4 levels (Fig. 4H-J). Consistently, cellular and extracellular DPP4 enzyme activity was decreased by these chemotherapeutic drugs (Fig. 4K, L). So chemotherapy downregulated total DPP4 in breast cancer possibly through inhibiting protein expression rather than by blocking enzyme activity. This speculation was confirmed in the tissue sample, and the proteome data showed that more DPP4 was enriched in the pre-chemotherapy compared with post-chemotherapy tumor tissue (Fig. 4M). In addition, we also explored the possibility of chemotherapy-releasing sICOSL through other mechanisms. ADAM10/17 protein levels showed no significant change in the breast cancer tissue after chemotherapy (Figure S3F). Paclitaxel also didn’t affect ADAM10/17 and ICOSL expression in breast cancer cell lines in vitro (Figure S3G). Therefore, we excluded the possibility that chemotherapy induced sICOSL release by increasing ADAM10/ADAM17 or ICOSL expression. In addition, DPP4 and ICOS expression were positively correlated in both breast cancer cohorts (Fig. 4N). Conclusively, it’s confirmed that cytotoxic drugs such as paclitaxel induced DPP4 expression in the tumor cells to block the degradation of sICOSL and stabilize ICOS in T cells.

### EZH2 mediate chemotherapy-induced DPP4 downregulation

**Fig. 5:**
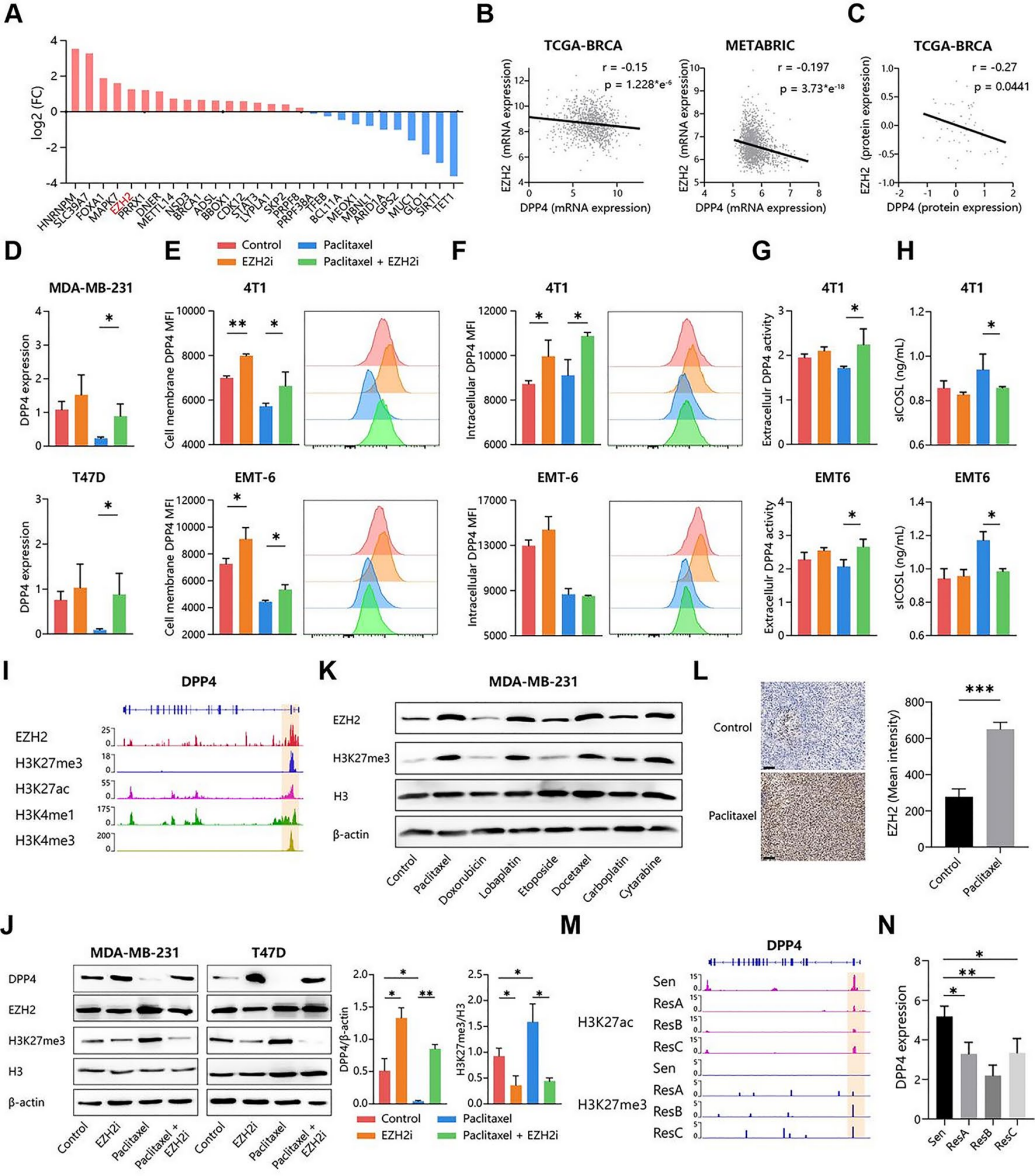
EZH2 mediate chemotherapy-induced DPP4 downregulation. (**A**) The regulators of DPP4 in breast cancer cell lines were identified based on GPSA datasets. The bar plot indicates fold change (FC) of DPP4 expression after inhibition of these regulators through knockdown/knockout/inhibitors. Positive (blue; FC < 1) and negative regulators (red; FC > 1) with p < 0.05 were shown. (**B**) The correlation between DPP4 and EZH2 expression in TCGA-BRCA and METABRIC datasets. (**C**) The correlation between DPP4 and EZH2 protein levels. (**D**-**H**) Several breast cancer cell lines were treated with paclitaxel and EZH2i (EZH2 inhibitor). DPP4 expression was measured through qPCR (**D**), flow cytometry (plasma membrane (**E**) and intracellular (**F**) staining) and enzyme activity assay (culture supernatant) (**G**). sICOSL concentration in culture supernatant was measured using ELISA (**H**). (**I**) Genomic visualization of EZH2, H3K27me3, H3K27ac, H3K4me1 and H3K4me3 signal intensity of DPP4 gene tracks. y-axis is normalized signal intensity. The DPP4 gene model is oriented right-to-left along the x axis. The DPP4 promoter was marked as orange zone. (**J**) DPP4, EZH2 and H3K27me3 protein levels of breast cancer cell lines after treated with paclitaxel and/or EZH2i. (**K**) EZH2 and H3K27me3 protein levels of MDA-MB-231 after treatment with different sICOSL-inducing chemotherapeutic drugs in vitro. H3 and β-actin were used as internal reference. (**L**) EZH2 protein levels of mammary tumors from paclitaxel-injected MMTV-PyMT mouse. Scale bar = 100 μm. (**M**, **N**) Deblois G et al., gerenated three paclitaxel-resistant model (ResA/B/C) from paclitaxel-sensitive (Sen) MDA-MB-436 cell lines and performed ChIP-seq and RNA-seq. Genomic visualization of H3K27ac and H3K27me3 signal intensity of DPP4 gene tracks (**M**) and DPP4 expression (**N**) in parental and resistant MDA-MB-436 cells

To determine the regulator of DPP4 expression in post-chemotherapy breast cancer, we performed the genetic perturbation similarity analysis (GPSA) based on the database (http://guotosky.vip:13838/GPSA/) and calculated the differential expression of DPP4 from gene knock down/out RNAseq datasets of breast cancer cell lines (Fig. 5A). We screened 29 regulators (11 positive and 18 negative) and verified them using correlation analysis between these genes and DPP4 in breast cancer cohort. Among all candidates, EZH2 (Enhancer Of Zeste 2 Polycomb Repressive Complex 2 Subunit) was significantly negatively correlated with DPP4 expression in both transcriptome and proteome datasets (Fig. 5B, C). Therefore, we speculated that EZH2 inhibited DPP4 expression in the breast cancer.

Then we tried to confirm the role of EZH2 in chemotherapy-induced DPP4-sICOSL axis. Breast cancer cell lines were treated with EZH2i (EZH2 inhibitor) and/or paclitaxel, the cytotoxic drug with the strongest ability to induce sICOSL release (Fig. 4A). Paclitaxel significantly decreased mRNA expression and protein levels of DPP4, which were reversed by EZH2i (Fig. 5D-G). Notably, EZH2i reduced DPP4 distributed in all subcellular locations, suggesting that EZH2 might regulate the transcription of DPP4 gene to control the total DPP4 protein (Fig. 5E-G). Relevantly, EZH2i also lowered the paclitaxel-induced sICOSL (Fig. 5H). In the absence of chemotherapy, EZH2i no longer significantly induced DPP4 expression, activity, and sICOSL levels, indicating that the EZH2-DPP4-sICOSL axis was fully effective only during chemotherapy (Fig. 5D-G). Correlation analysis showed that the sICOSL levels were mainly positively correlated with DPP4 in the cell membrane but not in the intracellular and extracellular spaces (Figure S4E). So membranous DPP4 were enzymetically active to degrade sICOSL after EZH2i and/or paclitaxel treatment. The reanalysis of a scRNA-seq dataset also showed that EZH2i increased almost the Dpp4 expression of all tumor-infiltrating immune cells (Figure S4A, B). Correspondingly, Icos + T cells also diminished after EZH2i treatment, indicating that EZH2 possibly mediate DPP4-sICOSL axis to maintain Icos stability in T cells (Figure S4C, D). In summary, EZH2 is the key regulator in chemotherapy-induced DPP4-sICOSL axis.

EZH2 serve as the enzymatically active core subunit of the Polycomb repressive complex 2 (PRC2), which catalyses histone H3 lysine 27 trimethylation (H3K27me3) to maintain a gene-repressive chromatin state [20]. The genome-binding datasets confirmed that the transcriptional start site (TSS) of DPP4 are directly cobound by EZH2 and epigenetic markers (H3K27me3, H3K27ac, H3K4me1 and H3K4me3),suggesting the role of EZH2 in DPP4 transcription (Fig. 5I). Consistently, EZH2 inhibitors also blockaded H3K27me3 and induced re-expression of DPP4, which was initially suppressed by paclitaxel in breast cancer cell lines (Fig. 5J). Therefore, it was concluded that EZH2 methylated H3K27 to silence DPP4 expression. In addition, high sICOSL-inducing drugs significantly promote EZH2 and H3K27me3 enrichment (Fig. 5K). Paclitaxel also increase EZH2 levels in the primay breast cancer and lung metastasis in vivo (Fig. 5L, S4F). In the other hand, In the other hand, compared to control cancer cells, in paclitaxel-resistant breast cancer, the H3K27me3 level at the DPP4 transcriptional start site (TSS) was higher, silencing DPP4 expression. This indicates that EZH2-mediated DPP4 downregulation also contributes to chemoresistance (Fig. 5M, N). In conclusion, some chemotherapeuatic drugs induce EZH2 expression to methylate the DPP4 TSS, which downregulate DPP4 transcription.

Considering the nonspecific nature of epigenetic regulation, there was possibly other bypass pathway to influence sICOSL release except for known EZH2-DPP4 axis. First, we detected the relationship between EZH2 and other known sICOSL-relavant genes, including ADAM10, ADAM17 and ICOSL. EZH2 inhibitor didn’t influence these gene expression (Figure S4G). EZH2 binding also wasn’t detected in the TSS of these genes (Figure S4H). EZH2-mediated epigenetic markers (ep. H3K27ac, H3K27me3) of these gene had no difference between paclitaxel-sensitive and resistant cell lines (Figure S4I). Therefore, EZH2 didn’t regulate transcription of other sICOSL-releasing genes. Second, we tried to discover other mechanism to regulate DPP4 expression besides EZH2. For instance, other epigenetic markers (ep. H3K4me1 and H3K4me3), although enriched in the DPP4 TSS (Fig. 5I), had no change between paclitaxel-sensitive and resistant cell lines (Figure S4J). Therefore, H3K4 methylation might have few effect in chemotherapy-related DPP4 regulation. Summarily, chemotherapy majorly mediates sICOSL release through the EZH2-DPP4 axis in the breast cancer microenvironments.

### sICOSL blockade therapy sensitizes tumors to chemoimmunotherapy

**Fig. 6:**
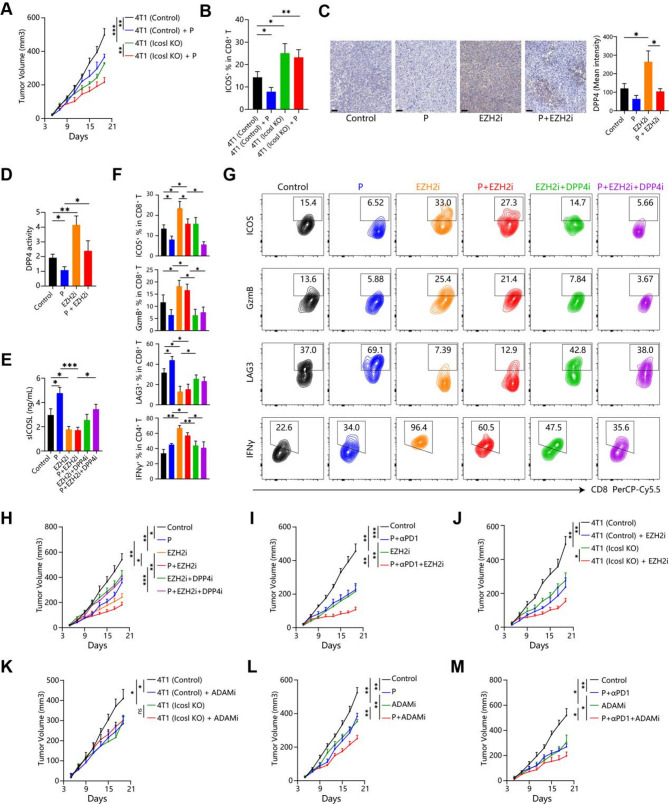
sICOSL blockade sensitizes tumors to chemoimmunotherapy. (**A**-**B**) BALB/c mice inoculated with syngeneic 4T1 breast cancer cells pretransduced with sgCtrl and sgICOSL, then injected with paclitaxel (P) (n = 8). (**A**) Tumor volume monitored every 3 days. (**B**) On day 20, mice were sacrificed, tumor-infiltrating lymphocytes isolated and analyzed via flow cytometry, showing ICOS positivity in CD8 + T cells. (**C**-**J**) BALB/c mice with syngeneic 4T1 breast cancer received P, EZH2 inhibitor (EZH2i), DPP4 inhibitor (DPP4i), and anti-PD1 antibody (αPD1). On day 20, mice were sacrificed to assess DPP4 protein levels (**C**) and enzymatic activity (**D**) in 4T1 mammary tumors (Scale bar = 100 μm), sICOSL concentration in tumor interstitial fluid (**E**), Granzyme B, LAG3, and IFNγ positivity in CD8 + T cells through quantitative histograms (**F**) and representative contour plots (**G**). (H-M) BALB/c mice bearing Icosl-KO or WT 4T1 breast cancer were injected with ADAM10/17 inhibitor (ADAMi), EZH2i, DPP4i, P, and/or αPD1. Tumor volume was monitored every 3 days (n = 8)

It was concluded that sICOSL mediates T cell dysfunction in the TME. Then we tried to inhibit sICOSL releasing through three methods to reverse the immune suppression in vivo. First, direct knockout of Icosl gene in cell lines considerably decreased serum and intra-tumoral sIcosl levels, and delayed tumor growth in vivo (Fig. 2F-G), but not in vitro (Figure S1B, D). Icosl-KO tumors were also more sensitive to paclitaxel than WT tumors (Fig. 6A, S5A). Consistently, fewer exhausted LAG3^+^CD8^+^ T cells were enriched in Icosl-KO tumors compared with WT tumors after paclitaxel treatment, potentially due to depleted sICOSL and thus stronger ICOS signaling (Fig. 6B, S5B). Therefore, sIcosl depletion using genetic perturbation enhances tumor response to chemotherapy.

Chemotherapy mainly induced EZH2 expression to inhibit DPP4 expression and upregulate downstream sICOSL levels. Consistently, EZH2i (EZH2 inhibitor) significantly decreased DPP4 protein levels and enzymatic activity in vivo (Fig. 6C, D, S5C). EZH2i also totally abolishes sICOSL releasing in paclitaxel-treated tumors, but this effect is reversed by DPP4i (Fig. 6E, S5D). Therefore, EZH2i modulated sICOSL levels through DPP4 in vivo, thus upregulated ICOS expression in CD8^+^ T cells (Fig. 6F, G), and could mediate response to chemotherapy. The tumor growth curves showed that EZH2i significantly sensitized tumors to chemotherapy (Fig. 6H, S5E), and also increased tumor response to combination therapy of paclitaxel and anti-PD1 antibody (Fig. 6I, S5F). Consistently, EZH2i promoted T cells activation (Granzyme B, IFNγ) and inhibited their exhaustion (LAG3) in tumor treated with chemotherapy (Fig. 6F, G), with similar phenomena as chemoimmunotherapy (Figure S5G). On the contrary, DPP4i weakened the immune-stimulatory effects of EZH2i (Fig. 6F, G), thereby diminishing their therapeutic efficacy (Fig. 6H, S5E). Correspondingly, EZH2 inhibitors exhibit some efficacy against Icosl-KO breast cancer, although this effect is weaker compared to that observed in wild-type breast cancer (Fig. 6J). In summray, EZH2 inhibitor enhances tumor response to chemoimmunotherapy through DPP4-sICOSL pathway.

The former literature and our results confirmed that ADAM10/17 shed membranous ICOSL to release the soluble form (Fig. 4C) [18]. Therefore, ADAM10/17 inhibitors (ADAMi) also decreased sICOSL levels in the tumor and serum (Figure S5H). ADAMi had no effects to Icosl-KO tumors growth but significantly curbed the WT tumors (Fig. 6K, S5I). Similarly, ADAMi only promoted T cells activation (ICOS, IFNγ) and inhibited their exhaustion (LAG3) in WT but not Icosl-KO tumors (Figure S5J), suggesting that ADAM10/17 mainly mediated TME through shedding ICOSL but not other target. Similar to EZH2 inhibitors, ADAMi also reversed T cell dysfunction and sensitized tumors to chemotherapy alone or chemoimmunotherapy (Fig. 6L, M, S5K, L).

In conclusion, sICOSL-blockade therapy, including EZH2 and ADAM10/17 inhibitors, enhanced T cell cytotoxicity, delayed tumor progression and enhanced tumors response to chemoimmunotherapy.

### Nanobody-DPP4 fusions enable targeted sICOSL degradation

**Fig. 7:**
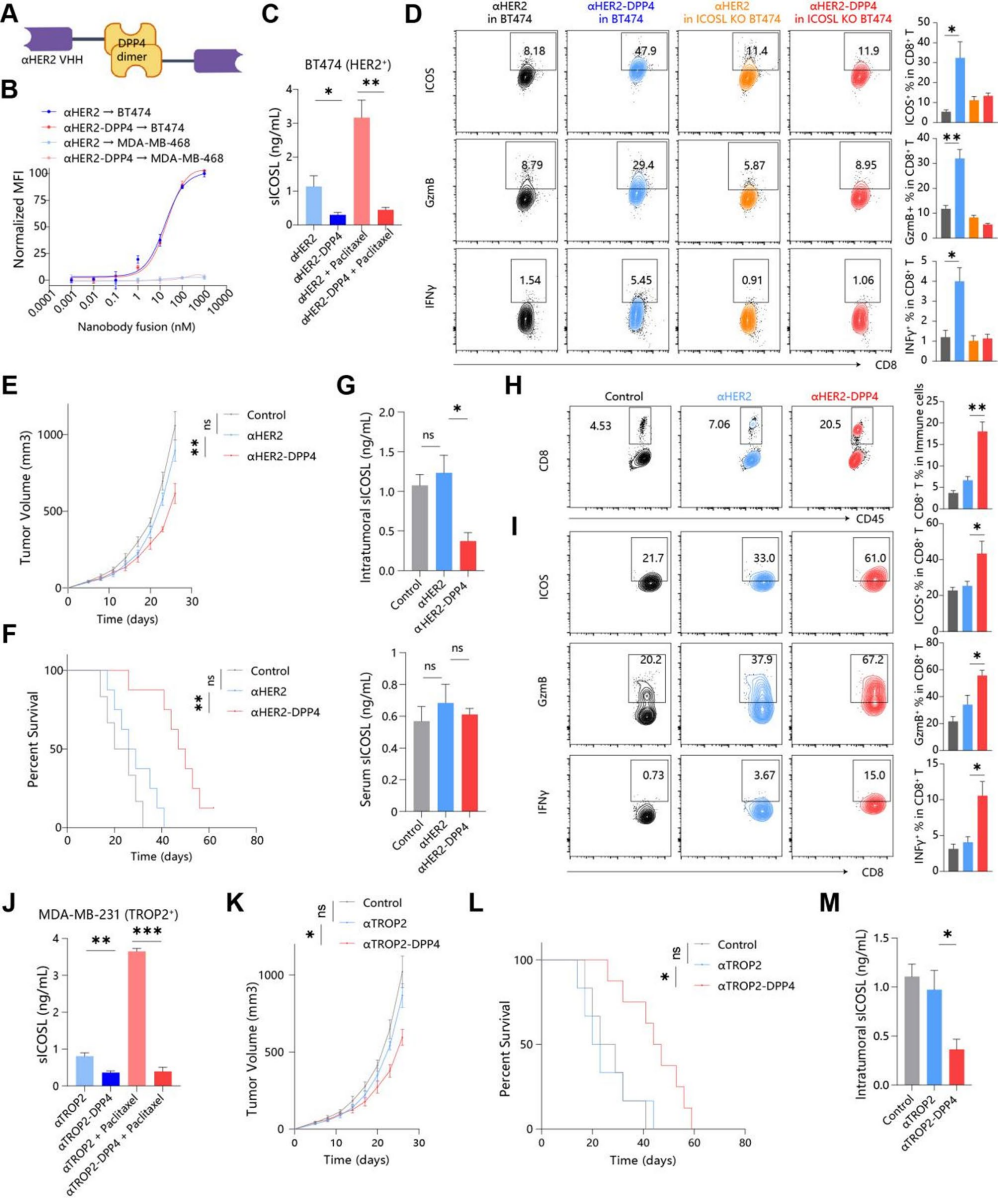
Nanobody-DPP4 fusions enable targeted sICOSL degradation. (**A**) Schematic of targeting sICOSL for enzymatic degradation via DPP4 in HER2+ cancers. (**B**) Binding curves of αHER2 and αHER2-DPP4 on HER2+ BT474 and HER2− MDA-MB-468 cells quantified by anti-His mean fluorescence intensity (MFI). (**C**) sICOSL concentration in the culture supernatant of HER2+ BT474 with αHER2/αHER2-DPP4 and chemotherapy in vitro. (**D**) Tumor-antigen activated CD8+ T cells were co-cultured with control and ICOSL KO BT474 cells and treated with αHER2 or αHER2-DPP4 for 18 h. ICOS, Granzyme B and IFNγ were stained in CD8+ T cells. Black: αHER2 in BT474; Blue: αHER2-DPP4 in BT474; Yellow: αHER2 in ICOSL KO BT474; Red: αHER2-DPP4 in ICOSL KO BT474. (E-H) BALB/c mice inoculated with syngeneic 4T1 breast cancer cells pretransduced with human HER2, and then were injected with αHER2 or αHER2-DPP4 (10 mg/kg i.v. q3d). Black: Control; Blue: αHER2; Red: αHER2-DPP4. Tumor volume (**E**) and mouse survival (**F**) were monitored every 3 days (n = 8). (**G**) sICOSL concerntration in the interstitial fluid of 4T1 mammary tumors and serum after treatment. Quantative histogram and representative contour plots showing CD8 + T infiltration (**H**) and activation marker (**I**) ICOS, Granzyme B and IFNγ positivity in CD8 + T cells. (**J**) sICOSL concerntration in the culture supernatant of TROP2+ MDA-MB-231 with αTROP2/αTROP2-DPP4 and chemotherapy in vitro. (K-M) BALB/c mice inoculated with syngeneic 4T1 breast cancer cells pretransduced with human TROP2, and then were injected with αTROP2 or αTROP2-DPP4. Tumor volume (**K**) and mouse survival (**L**) was monitored every 3 days (n = 8). (M) sICOSL concerntration in the interstitial fluid of 4T1 mammary tumors after treatment

sICOSL-blockade therapy only downregulated chemotherapy-induced sICOSL, but not influenced the baseline levels. In addition, these drugs, such as EZH2 and ADAM10/17 inhibitors, do not have tissue specificity which could lead to severe adverse effects. Therefore, we sought to develop a drug that can directly degrade intratumoral sICOSL. Given that DPP4 is the key degradation enzyme of sICOSL, we created a genetic fusion of DPP4 enzymes and antibodies targeting cell-surface receptor of breast cancer (Fig. 7A). Nanobodies rather than Fc-containing intact antibodies were applied to avoid degradation of membranous ICOSL in FcR expressing cells, such as dendritic cells (DC) and B cells [21] (Fig. 7A). HER2 was selected as an appropriate target antigen in breast cancer based on previous FDA-approved drugs [22]. Thus we designed conjugates with DPP4 C-terminal to the nanobody, which referred to as ‘αHER2-DPP4’. αHER2-DPP4 maintains as a homodimeric form (Fig. 7A, S6A), under which DPP4 exhibits enzymatic activity as reports [19]. αHER2-DPP4 significantly binds to BT474 (high HER2 expression) but not MDA-MB-468 (no HER2 expression) (Fig. 7B). Effective dissociation constants for αHER2-DPP4 (Kd = 21.5 nM) were comparable to that of puro nanobody (Kd = 16.5 nM), indicating that the fusion did not affect the affinity to target cells (Fig. 7B). Next, we tested the conjugate’s on-target activity in multiple human cancer cell lines and immune cells with different expression levels of HER2. 1 nM αHER2-DPP4 resulted in nearly complete loss of soluble ICOSL released by HER2^+^ cancer cells with or without paclitaxel treatment (Fig. 7C). and no discernable loss of sICOSL on HER2– cancer cells (Figure S6B). Therefore, nanobody-DPP4 fusions specifically target cancer cells to degrade sICOSL.

Using a series of cell assays, we tested whether nanobody-DPP4 could selectively reverse sICOSL-dependent CTL dysfunction. HER2 + breast cancer cells were treated with αHER2-DPP4 or αHER2, and cocultured with CTLs. αHER2-DPP4 upregulates ICOS, IFNγ, and GZMB expression of CTLs (Fig. 7D, S6C). Consistently, αHER2-DPP4 had few effects on CTLs when cocultured with ICOSL-knockout cell lines (Fig. 7D, S6C). Therefore, nanobody-DPP4 fusions degrade cancer-derived sICOSL to reverse CTL dysfunction. To assess the cytotoxicity of nanobody-DPP4 fusions, we treated breast cancer cells with varying HER2 expression levels with nanobody-DPP4 fusions in vitro. We observed that only at very high concentrations did the fusions significantly inhibit the proliferation of HER2-high breast cancer cells, with no effect on other cell lines (Figure S6D). This is consistent with other data showing that directly targeting ICOSL in cancer cells does not affect their proliferation (Figure S1B-G). Collectively, these data confirm that nanobody-DPP4 fusions primarily exert their therapeutic effects by disrupting sICOSL-mediated suppressive immune microenvironments, rather than by directly impacting tumor proliferation.

To assess on-target efficacy of αHER2-DPP4 in vivo, we turned to previously validated mouse models of HER2 positive breast cancer. This model involved injection of 4T1 cells stably expressing human HER2 (hHER2) into the mammary fat pads of mice followed by intraperitoneal (i.p.) treatment with αHER2-DPP4, an equimolar dose of αHER2 or vehicle control. Treatment with αHER2-DPP4 resulted in reduced tumor size (Fig. 7E). After 32 d, all mice in the vehicle-treated group had reached a tumor burden requiring euthanasia, while treatment with αHER2-DPP4 extended mouse survival to 62 d (Fig. 7F). Treatment with αHER2 alone did not result in attenuation of tumor growth or prolonged survival (Fig. 7E, F). As far as safety was concerned, mice treated with αHER2 or αHER2-DPP4 did not exhibit weight loss over the course of the experiment, suggesting that treatment was well tolerated (Figure S6E). Off-target toxicity was not found in liver, spleen, kidney, heart and lung (Figure S6F).

For analysis of sICOSL degradation and immune infiltration within HER2 tumors, a separate set of animals was treated as described above with vehicle, αHER2, or αHER2-DPP4 and euthanized at day 10 after implantation. ELISA analysis of αHER2-DPP4-treated animals revealed a significant reduction of intratumoral sICOSL with minimal effects on serum sICOSL confirming that αHER2-DPP4 selectively depletes intratumoral sICOSL in vivo (Fig. 7G). Both intratumoral and serum sICOSL were not changed in animals treated with vehicle or αHER2 control (Fig. 7G). We next profiled the immune composition within tumors and found that increased infiltration of CD8^+^ T cells into tumors after αHER2-DPP4 therapy (Fig. 7H). Notably, CD8^+^ T cells of conjugate-treated animals also exhibited significantly increased levels of ICOS, granzyme B and perforin (Fig. 7I). These data, which support the modulation of the tumor immune microenvironment following αHER2-DPP4 treatment, align with the significance of sICOSL signaling in the tumor microenvironment. When treating HER2^+^ breast cancer mice with ICOSL knockout, it was found that αHER2-DPP4 lost its efficacy, indicating that αHER2-DPP4 primarily exerts its therapeutic effects by targeting and degrading sICOSL (Figure S6G, H).

To further demonstrate the broad applicability of this therapy, we utilized TROP2 as a tumor antigen, which is highly expressed in triple-negative and luminal breast cancers [23], to construct the ‘αTROP2-DPP4’ fusion protein. Accordingly, we validated that it could degrade sICOSL released by TROP2^+^ cancer cells MDA-MB-231 in vitro (Fig. 7J). Furthermore, when 4T1 cells overexpressing hTROP2 were implanted into the mammary fat pads of mice and treated with αTROP2-DPP4 and an equivalent amount of control antibody, αTROP2-DPP4 significantly inhibited tumor proliferation (Fig. 7K) and extended the survival of the mice (Fig. 7L), while also mediating a significant degradation of intratumoral sICOSL (Fig. 7M). In summary, targeted degradation of tumor-derived sICOSL limits the progression of breast cancer.

### Prognosis value of sICOSL regulators

sICOSL has been proved to proved to mediate immune evasion after chemoimmunotherapy and identified as a potential drug target in cellular and animal experiments. However, it remains to be unclear about the impact of sICOSL in patients. Given that membraneous ICOSL (mICOSL) is highly expressed in both immune and cancer cells, it’s difficult to discriminate sICOSL and mICOSL in bulk tumors. Several relevant regulators, such as EZH2, DPP4, ADAM10, and ADAM17, have been confirmed to be significantly correlated with sICOSL levels in animal experiments (Fig. 6E, S4E, S5D, S5H) and human samples (Figure S7A). Therefore, we indirectly indicated sICOSL levels by detecting expression of these regulators and revealed the relationship between sICOSL releasing and patients’ prognosis.

We performed the survival analysis in the large breast cancer cohorts, such as The Cancer Genome Atlas (TCGA) and Molecular Taxonomy of Breast Cancer International Consortium (METABRIC) [24]. Breast cancer patients with high EZH2, ICOSLG expression and low DPP4 expresssion had potentially stronger ability to release sICOSL, and thus a significant adverse overall survival (Figure S7B). sICOSL could induce ICOS downregulation, which also related with poor prognosis (Figure S7B). Other than breast cancer, sICOSL-releasing signature might also correlate with outcomes of other cancer histologies. Based on TCGA pan-cancer cohorts, there are 7 kinds of histology in which EZH2-DPP4 axis were enriched in the tumors compared with normal samples, including BRCA (breast cancer), LIHC (liver hepatocellular carcinoma), LUAD (Lung adenocarcinoma), MESO (Mesothelioma), SARC (sarcoma), THCA (thyroid cancer) and UCEC (Figure S7C). Consistently, an enhanced EZH2-DPP4 axis and loss ICOS expression also indicated poor prognosis in these cancers (Figure S7D).

sICOSL-blockade therapy was confirmed to enhance tumor response to chemoimmunotherapy. So sICOSL regulators might also predict efficacy of chemoimmunotherapy. Paclitaxel-resistant breast cancer cell lines had increased EZH2, ADAM10, ADAM17, and ICOSL expression, suggesting high sICOSL-release ability (Figure S8A). Based on proteome data of the breast cancer cohort, tumors sensitive to chemotherapy expressed higher levels of DPP4, indicating less ability to degrade sICOSL (Figure S8B). Then reanalysis of two breast cancer RNA-seq datasets revealed that high sICOSL-release ability associated with worse survival after chemotherapy (Figure S8C) [25, 26]. In addition, immunohistochemistry (IHC) also confirmed that EZH2-DPP4 axis was enhanced in breast cancer resistant to chemotherapy (Figure S8D-E). In summary, sICOSL enrichment indicated non-response to chemotherapy. As for patients treated with immunotherapy, we reanalyzed two relevant RNA-seq datasets including clear cell renal cell carcinoma and melanoma [27, 28]. We found that high expression of pro-sICOSL genes (ep. EZH2, ADAM10) was associated with worse survival (Figure S8F). On the other side, DPP4 indicated better survival (Figure S8F). In addition, we performed IHC in tumor samples from a Non-small-cell lung carcinoma (NSCLC) cohort treated with anti-PD1 antibody treated (Figure S8G, H). Similar to the previously studied chemotherapy cohort, EZH2-DPP4 axis was enhanced in NSCLC resistant to immunotherapy. In conclusion, these sICOSL-related gene signature indicated the prognosis and chemoimmunotherapy efficacy of cancer patients.

## Discussion

The efficacy of chemotherapy is closely related to the TME. Chemotherapeutic agents have been shown to stimulate the adaptive immune system through several mechanisms, including inducing immunogenic cell death, transient lymphodepletion and direct stimulating immune cells [8]. However, it was also reported that specific subsets of cancer cells mediate an immunosuppressive microenvironment during chemotherapy [29–31]. Our data indicated that tumors treated with chemotherapy can directly release soluble proteins to induce CTL dysfunction. We identified sICOSL as the key inducing factor that inhibits CTL infiltration and induces their dysfunction.

ICOS-ICOSL axis played a key role in the generation of effector T cells in tumor etiology [32, 33]. Lu Y et al. reported that ICOSL in B cells boosted chemosensitivity by enhancing tumor-specific CD8 + T cells and the Th1/Treg ratio [34]. In contrast with membranous-bound ICOSL, soluble ICOSL induces the internalization and inactivation of ICOS in CTLs, thus mediating CTL dysfunction during chemotherapy. Immune checkpoint blockade (ICB) therapy can partially reverse the adverse effects of chemotherapeutic agents on CTL. Consistently, several phase III clinical trials found that ICB in combination with chemotherapy improved survival in breast cancer, leading to their approval as a first line treatment [35, 36]. sICOSL is a negative biomarker for chemoimmunotherapy and a potential targets for combination therapy.

Some chemotherapeutic agents, such as taxanes, anthracyclines and platinum, strongly induces sICOSL release through enhancing EZH2-DPP4 axis. EZH2 was reported to drive resistance to chemotherapy and immunotherapy by epigenetically silencing downstream genes [37–40]. We identified the new mechanism about how EZH2 mediated tumor immune evasion, and also validated the effective combination of EZH2 inhibitors and chemoimmunotherapy. Clinical trials also confirmed that EZH2i (tazemetostat) in combination with the anti-PD-1/PD-L1 immunotherapy drug, pembrolizumab, resulted in durable responses in advanced urothelial carcinoma (UC) and diffuse large B-cell lymphoma (DLBCL) patients [41, 42]. However, the lack of tumor specificity in EZH2 inhibitors results in off-target adverse effects, thereby constraining their clinical utility. In addition, EZH2i can also interfere with other signaling pathways rather than DPP4-sICOSL. Therefore, there is a need to refine therapeutic approaches to directly intervene with intratumoral sICOSL.

Targeted protein degradation (TPD) is an emerging strategy for the elimination of classically undruggable proteins [43]. Classically, TPD uses bispecific molecules to traffic unwanted proteins to endogenous cellular proteolytic machinery for degradation. Traditional TPD relies on suitable targets that are highly expressed on the membrane of cancer cells and capable of being internalized and degraded upon binding [43, 44]. In this study, we developed a strategy for degradation of sICOSL through exogenous enzymes without the need for endogenous degradation machinery. DPP4 is identified as a pivotal enzyme in regulating sICOSL levels in breast cancer. Fusing DPP4 with an anti-tumor-associated antigens nanobody enables the specific degradation of intratumoral sICOSL, prevents off-target side effects, but could be hard to penetrate into the tumor due to large molecular weight. Future efforts should focus on further optimizing the amino acid sequence of the DPP4 enzyme to reduce the protein’s molecular weight and enhance its specificity for sICOSL.

Together, we expand the current model by proposing that ICOSL in different form plays a crucial role in anti-tumor immunity, and also devise a strategy targeting the degradation of sICOSL for immunotherapy. This study opens the door to future investigations into the biogenesis and function of the sICOSL across cellular biology in normal development and disease.

## Method

### Cell culture

All cells were grown in a 37℃ incubator with 5% CO2. Human (MDA-MB-231, T47D) and mouse (4T1, EMT-6) breast cancer cell lines were obtained from the American Type Culture Collection. The cells tested negative for mycoplasma contamination and were authenticated by short tandem repeat profiling before use. These cells were cultured in RPMI 1640 or DMEM medium containing 10% fetal bovine serum. Cells were treated with sICOSL (11559-H08H; Sino Biological).

Blood from healthy donors were obtained from the Wuhan Union Hospital with IRB approval by the Ethical Committee of the Tongji Medical College of Huazhong University of Science and Technology. Informed written consent from all participants or next of kin was obtained prior to the research. Peripheral blood mononuclear cell (PBMC) were isolated from whole blood by density gradient centrifugation. DCs were generated as previously described with modifications [13]. PBMCs were cultured in RPMI1640 medium supplemented with GM-CSF (800U/mL; R&D) and IL-4 (400U/mL; R&D) for 6 days, were matured through incubation with LPS (100ng/ml; Sigma) and then pulsed with tumor-cell lysates. To generate tumor-specific CTLs, we isolated CD8^+^ T cells from PBMCs (Stemcell, 17853), and then incubated them with antigen-specific DCs (5:1) in RPMI 1640 medium supplemented with 25 U/ml IL-2 (Peprotech) for 6 days.

### Protein production and purification

The amino acid sequences for the αHER2 nanobody (previously published [44]), αTROP2 (previously published [45]), and DPP4 (uniprot P27487) were reverse translated, optimized for expression in Expi293 with Codon Optimization Tool, and cloned into the pSecTag2A plasmid. Nanobody and nanobody-DPP4 fusions were expressed and purified from Expi293 cells using transient transfection (Expifectamine, Thermo Fisher Scientific). Enhancers were added 20 h after transfection. Cells were incubated for 5d at 37 °C and 8% CO2. Medium was then collected by centrifugation at 4,000 g for 20 min. Proteins were purified by Ni-NTA affinity chromatography, buffer exchanged into PBS containing 20% glycerol, concentrated and flash frozen for storage at − 80 °C. Purity and integrity of all proteins were assessed by SDS–PAGE. Pre- and post-freeze stability was assessed via UV-vis spectrophotometry as well as SDS–PAGE.

### Protein degradation analysis

Cells were plated in 6- or 12-well plates and grown to ~ 70% confluency before treatment. Medium was aspirated, and cells were treated with nanobody-DPP4 fusions or nanobodies in complete growth medium. After incubation at 37 °C for the designated amount of time, cells were washed with PBS, lifted with versene and collected by centrifugation at 300 g for 5 min at 4 °C. Samples were then tested by ELISA to quantify protein levels. For the CHX chase assay analysis, cells were plated in 12-well plates and treated with CHX (50 µg/mL) and/or DPP4 inhibitor. At specific time points, tumor cell culture supernatants were collected, and the sICOSL levels in the supernatants were determined using ELISA.

### Cell surface binding analysis

Cells were trypsinized, quenched with media, and transferred to a 96-well V-bottom plate. Cells were washed three times with PBS + 0.5% BSA + 5 mM EDTA (FACS buffer) and incubated with nanobody-DPP4 fusions or nanobodies for 30 min on ice. Cells were washed three times and incubated with 25 nM AF488-labeled anti-His. Flow cytometry was performed to evaluate cell surface binding.

### In vivo mouse study

C57BL/6, Balb/c and nude mice (Gempharmatech) were bred in a specific pathogen-free facility, and female mice were utilized at 6–8 weeks of age. B6.FVB-Tg (MMTV-PyMT) mice were purchased from the Jackson Laboratory. All animal studies were performed under Institutional Animal Care and Use Committee-approved protocols at Tongji Medical College of Huazhong University of Science and Technology. Mice were randomized at the beginning of each experiment and experiments were not blinded. For orthotopic transplantations, Wild type and EMT-6 or 4T1 cells with various Icosl expression (5 × 10^5^ cells) were trypsinized, washed, resuspended in PBS and injected in the fourth mammary fat pads on one flank of the mice. Tumour growth was monitored over time, by performing bilateral vernier caliper measurements every day and mean tumour volumes were calculated using the formula (length × width2 )/2. Treatments were initiated when tumours reached approximately 100 mm3 (approximately 10 days after tumour induction), At various time points (noted in the relevant figure legends) after tumour inoculation, the mice were euthanized. Paclitaxel (Hengrui, 10 mg/kg), EZH2i (Tazemetostat, MedChemExpress, 20 mg/kg), DPP4i (Trelagliptin, MedChemExpress, 1 mg/kg), ADAM10/17i (TAPI-1, MedChemExpress, 8 mg/kg) and anti-mouse PD1 (BioXCell BE0146, 15 mg/kg) were injected intraperitoneally (i.p.). To assess the in vivo effects of Nanobody-DPP4 fusions, mice were intravenously injected with purified αHER2, αHER2-DPP4, αTROP2, and αTROP2-DPP4, each at a dose of 10 mg/kg, administered every three days. Animals were monitored daily; tumors were measured as previously described, and mouse weight was assessed throughout the study. Animals were euthanized upon reaching humane endpoints: loss of 20% of body weight, breathing impairment, or poor response to external stimuli. No signs of animal suffering or discomfort were observed during the experiment. For survival monitoring, each mouse was sacrificed individually when tumors reached 1,000 mm³.

Mice were sacrificed when any diameter reached 15 mm. For functional experiments, tumors were collected and processed at the time points indicated in figure legends. Mouse tumors were mechanically disrupted using scissors, digested with a mixture of 0.5 mg/ml DNase (Sigma-Aldrich) and 1 mg/ml Collagenase IV (Sigma-Aldrich) in serum-free RPMI for 30 min. The single-cell suspension of tumors was dispersed through a 70-µm filter. Erythrolysis of whole blood and spleen samples were performed using the BD Pharm Lyse buffer (BD).

### Patient data and study approval

FFPE surgery samples from patients with BRCA and NSCLC were obtained from the Wuhan Union Hospital according to IRB-approved protocols. For BRCA patients, they received neoadjuvant chemotherapy and then surgery. The response was evaluated based on residual cancer burden (RCB) score. For NSCLC patients, they received surgery at Wuhan Union Hospital and experienced local recurrence or distant metastasis during postoperative follow-up. Then, they underwent anti-PD-1 treatment for recurrent unresectable or stage IV disease. Tumor samples were collected at baseline according to a standard pathology procedure. The response was defined as achieving a complete or partial radiographic response by iRECIST between pre-treatment imaging and post-treatment imaging. Target diseases, including lung and metastatic sites, were measured based on radiologic imaging, such as CT, MRI, and PET/CT. Patients information was provided before [46].

### Flow cytometry

T cells were treated in vitro as indicated at 37℃. The following fluorophore-conjugated antibodies were used: anti-human CD3 (BD; SK7), CD4 (Biolegend; RPA-T4), CD8 (Biolegend; SK1), Granzyme B (Biolegend; QA16A02), IFNγ (BD; 4 S.B3), TIM3 (Biolegend; F38-2E2), ICOS (Biolegend; C398.4 A), LAG3 (Biolegend; 11C3C65). For flow cytometry analysis of patient samples, single-cell suspensions were prepared from surgical specimens of breast cancer patients who had received neoadjuvant therapy. The cells were incubated with Fixable Viability Stain 510 or 780 (BD Horizon) for 30 min at 4 °C. They were then stained with antibodies against surface markers, including anti-human CD3 (BD; SK7), CD4 (Biolegend; RPA-T4), CD8 (Biolegend; SK1), CD45 (Biolegend; HI30), ICOS (Biolegend; C398.4 A), PD-1 (Biolegend; RMP1-30), PD-L1 (Biolegend; 29E.2A3), FAS (Biolegend; DX2), EPCAM (BD; EBA-1), CD31 (Biolegend; WM59), and CD68 (Biolegend; Y1/82A). The antibodies were incubated for 30 min at 4 °C. Subsequently, cell fixation and permeabilization were performed using IC fixation buffer (Invitrogen, FB001) and permeabilization buffer (Invitrogen, 00-8333-56). Cells were then stained with perforin (Biolegend; B-D48) in 1× permeabilization buffer at room temperature for 1 h. Finally, the cells were analyzed by multicolor flow cytometry using an FACS LSRII (BD Biosciences).

For in vivo breast cancer model, samples were stained with Fixable Viability Stain 510 or 780 (BD Horizon) and fluorescent dye-conjugated antibodies anti-mouse CD45 (HI30), CD3 (BD; ), CD4 (BD; RM4-5), CD8 (Biolegend; 53–6.7), IFNγ (Biolegend; XMG1.2), Granzyme B (Biolegend; QA16A02), ICOS (Biolegend; 15F9), LAG3 (Biolegend; C9B7W), EPCAM (Biolegend; G8.8), CD31 (Biolegend; 390), CD19 (BD; 1D3), CD68 (BD; FA/11), CD11C (BD; B-Iy6), NKp46 (Biolegend; 29A1.4), Ly6C (Biolegend; HK1.4), F4/80 (Biolegend; BM8), DPP4 (Biolegend; H194-112). To analyze DPP4 expression in different clusters, cells from MMTV-PyMT models were gated as Tumor (EPCAM^+^CD45^−^), CD4 + T (CD45^+^CD3^+^CD8^−^NKp46^−^), CD8 + T (CD45^+^CD3^+^CD8^+^NKp46^−^), NKT (CD45^+^CD3^+^NKp46^+^), NK (CD45^+^CD3^−^NKp46^+^), B (CD45^+^CD19^+^), Macrophage (CD45^+^F4/80^+^), M-MDSC (CD45^+^F4/80^−^Ly6C^hi^), DC (CD45^+^F4/80^−^CD11c^+^), CAF (EPCAM^−^CD45^−^CD31^−^), Endothelial (EPCAM^−^CD45^−^CD31^+^) Anti-human and mouse CD16/CD32 (BD; 2.4G2) were used as the blocking reagent to reduce the non-specific binding of the antibodies. For intracellular staining of IFNγ, Cytofix/Cytoperm solution (BD) was added before fixation and permeabilization. We performed the acquisition with FACS LSRII (BD Biosciences) and data analysis in FlowJo software (FlowJo LLC, Ashland, Oregon, USA). Flow cytometry graphs shown in the results section were representative data from at least three independent experiments.

### Cytotoxicity assays

Breast cancer cells were stained with CellTrace (Thermo Fisher Scientific, C34557) for 20 min at 37℃, and cocultured with the tumor-specific CTLs at an effector/target ratio of 10:1 for 12 h at 37 °C. After 12 h, all cells were harvested and stained with zombie NIR Fixable Viability Kit (Biolegend) and analyzed by flow cytometry.

### Immunoblot

Whole-cell lysates were prepared as previously described [14]. Equal amounts of protein (10–50 µg) were resolved by 12% SDS-PAGE. After electrophoresis, separated proteins were transferred onto the nitrocellulose membrane. The membrane was blocked in 5% non-fat milk, followed by overnight incubation with primary antibodies. After incubation with HRP-conjugated secondary antibody, the positive immune reactive signal was detected by ECL (Fude Biotech, Hangzhou, China). Antibodies specific for β-actin (sc-47778, 1:1000) were purchased from Santa Cruz Biotechnology (Santa Cruz, CA, USA). Antibodies specific for DPP4 (ab215711, 1:1000) and H3K27me3 (ab192985, 1:1000) were obtained from Abcam (Cambridge, UK). Antibodies specific for EZH2 (5246, 1:1000) were obtained from Cell Signaling Technology (Danvers, MA, USA). Antibodies specific for H3 (17168-1-AP 1:1000) were purchased from Proteintech.

### Immunohistochemistry and immunofluorescence

Immunohistochemistry staining was performed on FFPE tumor tissue sections. The tumor tissues were fixed in 10% formalin, embedded in paraffin, and serially sectioned for 3 μm thick. The following primary antibodies were used: DPP4 (ab215711, 1:100) and EZH2 (5246, 1:100). Immunohistochemical staining was performed according to the manufacturer’s instructions. Immunofluorescence staining was performed on T cells. For immunofluorescence multiplex staining, we followed the staining method for the following markers: anti-ICOS with fluorescein FITC (1:50), LysoTracker Deep Red, Dil and nuclei visualized with DAPI. A Nikon Ti-E microscope was used for all imaging. Image analysis was performed using NIS software modules (Nikon, version 4).

### Transcriptome

Transcriptome was performed on breast cancer samples with the TruSeq Stranded mRNA Preparation Kit. Libraries were pooled, and sequencing runs were performed in paired-end mode using the Illumina HiSeq 4000 platform. Initial RNAseq quality control (QC) checks included an evaluation of GC and per-base sequence content using FastQC (v0.11.5). All samples that passed initial QC were aligned to the human genome assembly (build hg19) using the STAR (v2.5) two-pass method with quantMode parameters set to TranscriptomeSAM for alignments translated into transcript coordinates. Alignments were sorted with SAMTools (v1.3.1), duplicates were marked with Picard Tools (v2.4.1), reads were split and trimmed, and mapping qualities were reassigned with the Genome Analysis Toolkit (v3.6) using the methods SplitNCigarReads and ReassignOneMappingQuality, respectively. Post-alignment QC required at least 70% of reads to be uniquely aligned, assessed using STAR alignment statistics (100% of samples passed). All RNAseq expression values were represented as transcripts per kilobase million (TPM).

Quantative proteomicsTandem Mass Tag (TMT) Liquid-Chromatography/Mass-Spectrometry (LC/MS) proteomics was completed as previously described [47]. Briefly, Around 1 ~ 1.5 mg FFPE tissue samples were processed to generate peptide samples using accelerated Pressure Cycling Technology (PCT) assisted sample preparation method. All peptide samples were labeled with TMTpro 16plex reagent (Thermo Fisher Scientific, USA). 16 TMT-labeled peptide samples were combined and desalted by C18 columns. Fractionation was performed on Thermo Ultimate Dinex 3000 (Thermo Fisher Scientific, San Jose, USA) with an XBridge Peptide BEH C18 column (Waters, Milford, MA, USA). Fractions were dried in vacuum, re-dissolved in 2% ACN/0.1% formic acid and then analyzed by a Q Exactive HF-X hybrid Quadrupole-Orbitrap. MS raw files were analyzed using the MaxQuant software and peptide lists were searched against the human Uniprot FASTA database. False discovery rate (FDR) was 0.01 for both the protein and peptide level with a minimum length of 7 amino acids for peptides and this FDR was determined by searching a reverse sequence database. Peptides were identified with an initial precursor mass deviation of up to 7 ppm and a fragment mass deviation of 20 ppm. Protein identification required at least one unique or razor peptide per protein group. Contaminants, and reverse identification were excluded from further data analysis.

### Bioinformatic analysis

scRNA-seq datasets of mouse tumor-infiltrating CD45^+^ Cells treated with EZH2 inhibitor from GSE188473 were analyzed. The Seurat package was used to filter out bad quality cells and normalize counts [48]. Count data in the downstream analysis were already normalized and log2 transformed. For all cells in scRNA-seq profiles, clusters were annotated based on the expression of known marker genes. These annotations were also confirmed by identifying differentially expressed marker genes for each cluster and comparing them to known cell-type-specific marker genes. After cell cluster annotation above, single cells from tumors were projected to 2D space using the UMAP with the color indication for Dpp4 and Icos expression.

### Statistical methods

Unless otherwise stated, the Mann Whitney U test was used to assess for a difference in distributions between two population groups. Survival analysis was conducted using a Cox proportional hazards model. Statistical analysis was carried out using R4.0.1 (http://www.r-project.org/) or greater. We considered a p-value of 0.05 as being statistically significant.

## Electronic supplementary material

Below is the link to the electronic supplementary material.

Supplementary Material 1

Supplementary Material 2

## Data Availability

No datasets were generated or analysed during the current study.

## References

[1] Siegel RL, Giaquinto AN, Jemal A. Cancer statistics, 2024. CA Cancer J Clin. 2024;74(1):12–49. 38230766 10.3322/caac.21820

[2] Vaidya JS, Massarut S, Vaidya HJ, Alexander EC, Richards T, Caris JA, et al. Rethinking neoadjuvant chemotherapy for breast cancer. BMJ. 2018;360:j5913. 29326104 10.1136/bmj.j5913

[3] Guo L, Kong D, Liu J, Zhan L, Luo L, Zheng W, et al. Breast cancer heterogeneity and its implication in personalized precision therapy. Exp Hematol Oncol. 2023;12(1):3. 36624542 10.1186/s40164-022-00363-1 PMC9830930

[4] Zheng S, Wang W, Shen L, Yao Y, Xia W, Ni C. Tumor battlefield within inflamed, excluded or desert immune phenotypes: the mechanisms and strategies. Exp Hematol Oncol. 2024;13(1):80. 39107856 10.1186/s40164-024-00543-1 PMC11301948

[5] Sharma P, Barlow WE, Godwin AK, Parkes EE, Knight LA, Walker SM, et al. Validation of the Dna damage immune response signature in patients with triple-negative breast cancer from the swog 9313c trial. J Clin Oncol. 2019;37(36):3484–92. 31657982 10.1200/JCO.19.00693 PMC7194448

[6] Casares N, Pequignot MO, Tesniere A, Ghiringhelli F, Roux S, Chaput N, et al. Caspase-dependent immunogenicity of doxorubicin-induced tumor cell death. J Exp Med. 2005;202(12):1691–701. 16365148 10.1084/jem.20050915 PMC2212968

[7] Galluzzi L, Buque A, Kepp O, Zitvogel L, Kroemer G. Immunogenic cell death in cancer and infectious disease. Nat Rev Immunol. 2017;17(2):97–111. 27748397 10.1038/nri.2016.107

[8] Bracci L, Schiavoni G, Sistigu A, Belardelli F. Immune-based mechanisms of cytotoxic chemotherapy: implications for the design of novel and rationale-based combined treatments against cancer. Cell Death Differ. 2014;21(1):15–25. 23787994 10.1038/cdd.2013.67 PMC3857622

[9] Nagarsheth N, Wicha MS, Zou W. Chemokines in the cancer microenvironment and their relevance in cancer immunotherapy. Nat Rev Immunol. 2017;17(9):559–72. 28555670 10.1038/nri.2017.49 PMC5731833

[10] Gu D, Ao X, Yang Y, Chen Z, Xu X. Soluble immune checkpoints in cancer: production, function and biological significance. J Immunother Cancer. 2018;6(1):132. 30482248 10.1186/s40425-018-0449-0 PMC6260693

[11] Kavanagh EL, Lindsay S, Halasz M, Gubbins LC, Weiner-Gorzel K, Guang MHZ, et al. Protein and chemotherapy profiling of extracellular vesicles harvested from therapeutic induced senescent triple negative breast cancer cells. Oncogenesis. 2017;6(10):e388. 28991260 10.1038/oncsis.2017.82 PMC5668881

[12] Watson MJ, Vignali P, Mullett SJ, Overacre-Delgoffe AE, Peralta RM, Grebinoski S, et al. Metabolic support of tumour-infiltrating regulatory t cells by lactic acid. Nature. 2021;591(7851):645–51. 33589820 10.1038/s41586-020-03045-2 PMC7990682

[13] Wu Z, Xi Z, Xiao Y, Zhao X, Li J, Feng N, et al. Tsh-tshr axis promotes tumor immune evasion. J Immunother Cancer. 2022;10(1):e4049. 10.1136/jitc-2021-004049 PMC8804696 35101946

[14] Wu Z, Xu Z, Zhou X, Li H, Zhao L, Lv Y, et al. Sgrp78 enhances selective autophagy of monomeric tlr4 to regulate myeloid cell death. Cell Death Dis. 2022;13(7):587. 35798718 10.1038/s41419-022-05048-5 PMC9262968

[15] Wang X, Tokheim C, Gu SS, Wang B, Tang Q, Li Y, et al. In vivo crispr screens identify the e3 ligase cop1 as a modulator of macrophage infiltration and cancer immunotherapy target. Cell. 2021;184(21):5357–74. 34582788 10.1016/j.cell.2021.09.006 PMC9136996

[16] Wikenheiser DJ, Stumhofer JS. Icos co-stimulation: friend or foe? Front Immunol. 2016;7:304. 27559335 10.3389/fimmu.2016.00304 PMC4979228

[17] Tafuri A, Shahinian A, Bladt F, Yoshinaga SK, Jordana M, Wakeham A, et al. Icos is essential for effective t-helper-cell responses. Nature. 2001;409(6816):105–9. 11343123 10.1038/35051113

[18] Lownik JC, Luker AJ, Damle SR, Cooley LF, El SR, Hutloff A, et al. Adam10-mediated Icos ligand shedding on b cells is necessary for proper t cell Icos regulation and t follicular helper responses. J Immunol. 2017;199(7):2305–15. 28814605 10.4049/jimmunol.1700833 PMC5605448

[19] Enz N, Vliegen G, De Meester I, Jungraithmayr W. Cd26/dpp4 - a potential biomarker and target for cancer therapy. Pharmacol Ther. 2019;198(135– 59. 10.1016/j.pharmthera.2019.02.015 30822465

[20] Kim KH, Roberts CWM. Targeting ezh2 in cancer. Nat Med. 2016;22(2):128–34. 26845405 10.1038/nm.4036 PMC4918227

[21] Amatore F, Gorvel L, Olive D. Role of inducible co-stimulator (icos) in cancer immunotherapy. Expert Opin Biol Ther. 2020;20(2):141–50. 31738626 10.1080/14712598.2020.1693540

[22] Cortés J, Kim S, Chung W, Im S, Park YH, Hegg R, et al. Trastuzumab Deruxtecan versus trastuzumab emtansine for breast cancer. N Engl J Med. 2022;386(12):1143–54. 35320644 10.1056/NEJMoa2115022

[23] Bardia A, Hurvitz SA, Tolaney SM, Loirat D, Punie K, Oliveira M, et al. Sacituzumab Govitecan in metastatic triple-negative breast cancer. N Engl J Med. 2021;384(16):1529–41. 33882206 10.1056/NEJMoa2028485

[24] Curtis C, Shah SP, Chin SF, Turashvili G, Rueda OM, Dunning MJ, et al. The genomic and transcriptomic architecture of 2,000 breast tumours reveals novel subgroups. Nature. 2012;486(7403):346–52. 22522925 10.1038/nature10983 PMC3440846

[25] Loi S, Haibe-Kains B, Desmedt C, Lallemand F, Tutt AM, Gillet C, et al. Definition of clinically distinct molecular subtypes in Estrogen receptor-positive breast carcinomas through genomic grade. J Clin Oncol. 2007;25(10):1239–46. 17401012 10.1200/JCO.2006.07.1522

[26] Bos PD, Zhang XHF, Nadal C, Shu W, Gomis RR, Nguyen DX, et al. Genes that mediate breast cancer metastasis to the brain. Nature. 2009;459(7249):1005–9. 19421193 10.1038/nature08021 PMC2698953

[27] Braun DA, Hou Y, Bakouny Z, Ficial M, Sant Angelo M, Forman J, et al. Interplay of somatic alterations and immune infiltration modulates response to pd-1 Blockade in advanced clear cell renal cell carcinoma. Nat Med. 2020;26(6):909–18. 32472114 10.1038/s41591-020-0839-y PMC7499153

[28] Gide TN, Quek C, Menzies AM, Tasker AT, Shang P, Holst J, et al. Distinct immune cell populations define response to anti-pd-1 monotherapy and anti-pd-1/anti-ctla-4 combined therapy. Cancer Cell. 2019;35(2):238–55. 30753825 10.1016/j.ccell.2019.01.003

[29] Zhang Y, Yu M, Jing Y, Cheng J, Zhang C, Cheng L, et al. Baseline immunity and impact of chemotherapy on immune microenvironment in cervical cancer. Br J Cancer. 2021;124(2):414–24. 33087896 10.1038/s41416-020-01123-w PMC7852680

[30] Samanta D, Park Y, Ni X, Li H, Zahnow CA, Gabrielson E, et al. Chemotherapy induces enrichment of cd47(+)/cd73(+)/pdl1(+) immune evasive triple-negative breast cancer cells. Proc Natl Acad Sci U S A. 2018;115(6):E1239–48. 29367423 10.1073/pnas.1718197115 PMC5819443

[31] Baldominos P, Barbera-Mourelle A, Barreiro O, Huang Y, Wight A, Cho J, et al. Quiescent cancer cells resist t cell attack by forming an immunosuppressive niche. Cell. 2022;185(10):1694–708. 35447074 10.1016/j.cell.2022.03.033 PMC11332067

[32] Peng C, Huggins MA, Wanhainen KM, Knutson TP, Lu H, Georgiev H, et al. Engagement of the costimulatory molecule Icos in tissues promotes establishment of cd8 + tissue-resident memory t cells. Immunity. 2022;55(1):98–114. 34932944 10.1016/j.immuni.2021.11.017 PMC8755622

[33] Metzger TC, Long H, Potluri S, Pertel T, Bailey-Bucktrout SL, Lin JC, et al. Icos promotes the function of cd4 + effector t cells during anti-ox40–mediated tumor rejection. Cancer Res. 2016;76(13):3684–9. 27197182 10.1158/0008-5472.CAN-15-3412

[34] Lu Y, Zhao Q, Liao J, Song E, Xia Q, Pan J, et al. Complement signals determine opposite effects of b cells in chemotherapy-induced immunity. Cell. 2020;180(6):1081–97. 32142650 10.1016/j.cell.2020.02.015

[35] Schmid P, Cortes J, Pusztai L, Mcarthur H, Kummel S, Bergh J, et al. Pembrolizumab for early triple-negative breast cancer. N Engl J Med. 2020;382(9):810–21. 32101663 10.1056/NEJMoa1910549

[36] Schmid P, Adams S, Rugo HS, Schneeweiss A, Barrios CH, Iwata H, et al. Atezolizumab and nab-paclitaxel in advanced triple-negative breast cancer. N Engl J Med. 2018;379(22):2108–21. 30345906 10.1056/NEJMoa1809615

[37] Gardner EE, Lok BH, Schneeberger VE, Desmeules P, Miles LA, Arnold PK, et al. Chemosensitive relapse in small cell lung cancer proceeds through an ezh2-slfn11 axis. Cancer Cell. 2017;31(2):286–99. 28196596 10.1016/j.ccell.2017.01.006 PMC5313262

[38] Fillmore CM, Xu C, Desai PT, Berry JM, Rowbotham SP, Lin YJ, et al. Ezh2 Inhibition sensitizes brg1 and Egfr mutant lung tumours to topoii inhibitors. Nature. 2015;520(7546):239–42. 25629630 10.1038/nature14122 PMC4393352

[39] Deblois G, Tonekaboni S, Grillo G, Martinez C, Kao YI, Tai F, et al. Epigenetic switch-induced viral mimicry evasion in chemotherapy-resistant breast cancer. Cancer Discov. 2020;10(9):1312–29. 32546577 10.1158/2159-8290.CD-19-1493

[40] Wen Y, Hou Y, Yi X, Sun S, Guo J, He X, et al. Ezh2 activates chk1 signaling to promote ovarian cancer chemoresistance by maintaining the properties of cancer stem cells. Theranostics. 2021;11(4):1795–813. 33408782 10.7150/thno.48101 PMC7778604

[41] Maha HA, Hussain MKPS. A pilot study of Tazemetostat and pembrolizumab in advanced urothelial carcinoma (etctn 10183). J Clin Oncol. 2023;6(41):suppl.

[42] Palomba ML, Cartron G, Popplewell L, Ribrag V, Westin J, Huw LY, et al. Combination of Atezolizumab and Tazemetostat in patients with relapsed/refractory diffuse large b-cell lymphoma: results from a phase Ib study. Clin Lymphoma Myeloma Leuk. 2022;22(7):504–12. 35151584 10.1016/j.clml.2021.12.014

[43] Wells JA, Kumru K. Extracellular targeted protein degradation: an emerging modality for drug discovery. Nat Rev Drug Discov. 2024;23(2):126–40. 38062152 10.1038/s41573-023-00833-z

[44] Pedram K, Shon DJ, Tender GS, Mantuano NR, Northey JJ, Metcalf KJ, et al. Design of a mucin-selective protease for targeted degradation of cancer-associated mucins. Nat Biotechnol. 2024;42(4):597–607. 37537499 10.1038/s41587-023-01840-6 PMC11018308

[45] Hu Y, Wang Y, Lin J, Wu S, Lv H, Ji X, et al. Identification and characterization of specific nanobodies against trop-2 for tumor targeting. Int J Mol Sci. 2022;23(14):7942. 35887287 10.3390/ijms23147942 PMC9316174

[46] Wu Z, Zhou J, Xiao Y, Ming J, Zhou J, Dong F, et al. Cd20 + cd22 + adam28 + b cells in tertiary lymphoid structures promote immunotherapy response. Front Immunol. 2022;13:865596. 35634306 10.3389/fimmu.2022.865596 PMC9130862

[47] Yu K, Wang Z, Wu Z, Tan H, Mishra A, Peng J. High-throughput profiling of proteome and posttranslational modifications by 16-plex Tmt labeling and mass spectrometry. Methods Mol Biol. 2021;2228:(205– 24. 10.1007/978-1-0716-1024-4_15 PMC8458009 33950493

[48] Stuart T, Butler A, Hoffman P, Hafemeister C, Papalexi E, Mauck WM, et al. Comprehensive integration of single-cell data. Cell. 2019;177(7):1888–902. 31178118 10.1016/j.cell.2019.05.031 PMC6687398

