# Supplementary material for: Targeted degradation of sICOSL reverses cytotoxic T cells dysfunction
Source: Exp Hematol Oncol. 2025 Jul 24;14:100. doi: 10.1186/s40164-025-00692-x (PMC12288325; doi:10.1186/s40164-025-00692-x)
Supplement: Supplementary file 2 — Supplementary Material 2 [file 40164_2025_692_MOESM2_ESM.docx]

Supporting Information

## **Targeted degradation of sICOSL reverses cytotoxic T cells dysfunction**

Zhenghao Wu, Peng Zheng, Ruobing Qi , Yunxiao Xiao, Zihan Xi, Lei Dai, Tao Chen, Rong Wang, Zimei Tang, Xiangwang Zhao, Jie Tan, Jie Ming, Ping Lei, Chunping Liu ^*^, Tao Huang ^*^


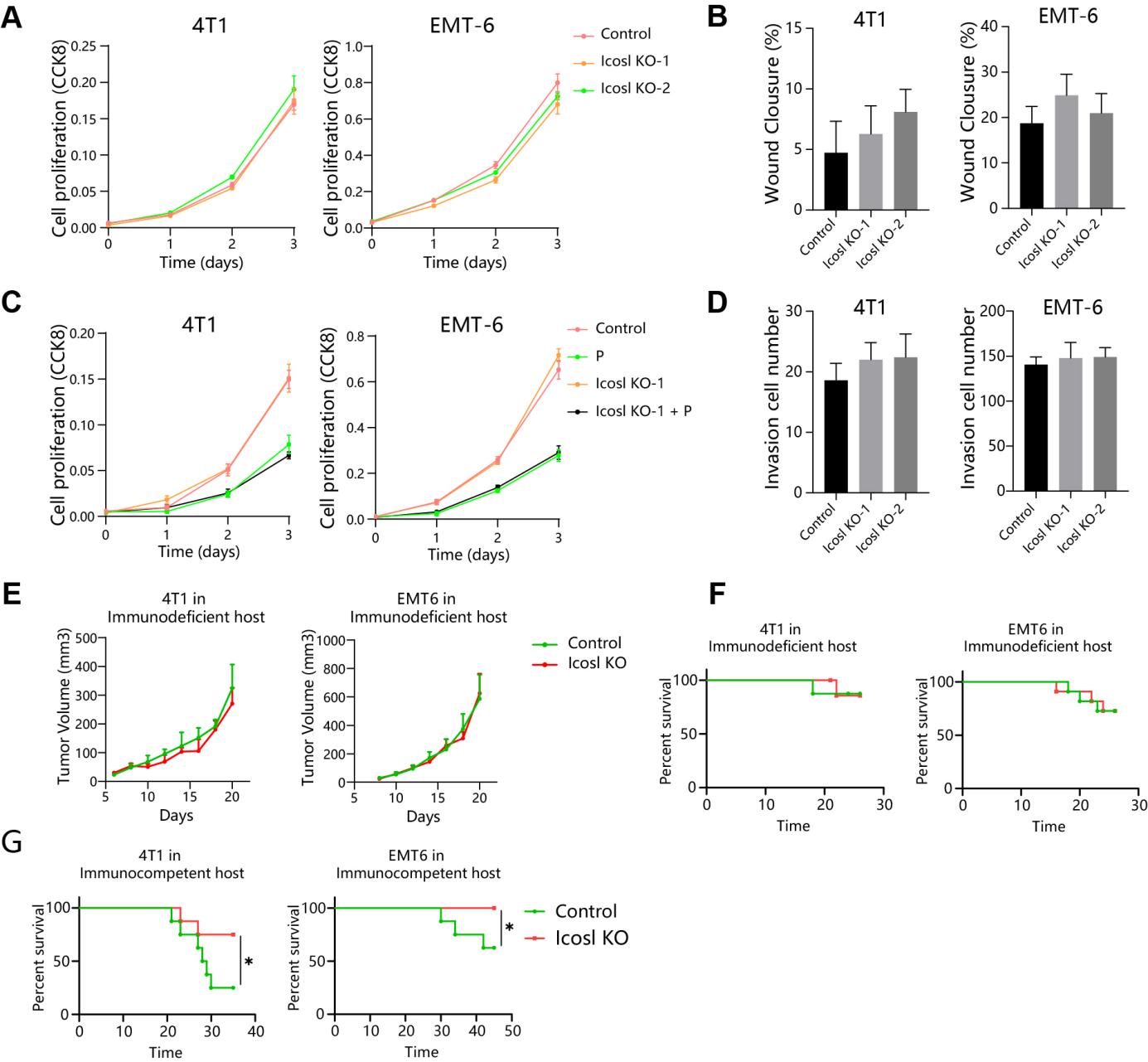


Figure S1, Differential effects of tumor Icosl-KO on tumor growth in vitro.

(A) Evaluation of Icosl-KO/WT 4T1 and EMT-6 cells proliferation through CCK-8 assay; (B) Evaluation of Icosl-KO/WT cells migratory abilities through wound healing assays; (C) Cell proliferation of Icosl-KO/WT cells treated with paclitaxel; (D) Evaluation of Icosl-KO/WT cells invasive capacities through transwell assays. Representative images of three independent experiments with similar results are shown. (E) Tumor growth of Icosl-KO/WT 4T1 and EMT-6 in immunodeficient mice (n = 8). (F, G) Survival curve of Icosl-KO/WT 4T1 and EMT-6 tumor growth in immunodeficient hosts (F) and immunocompetent mice (G).


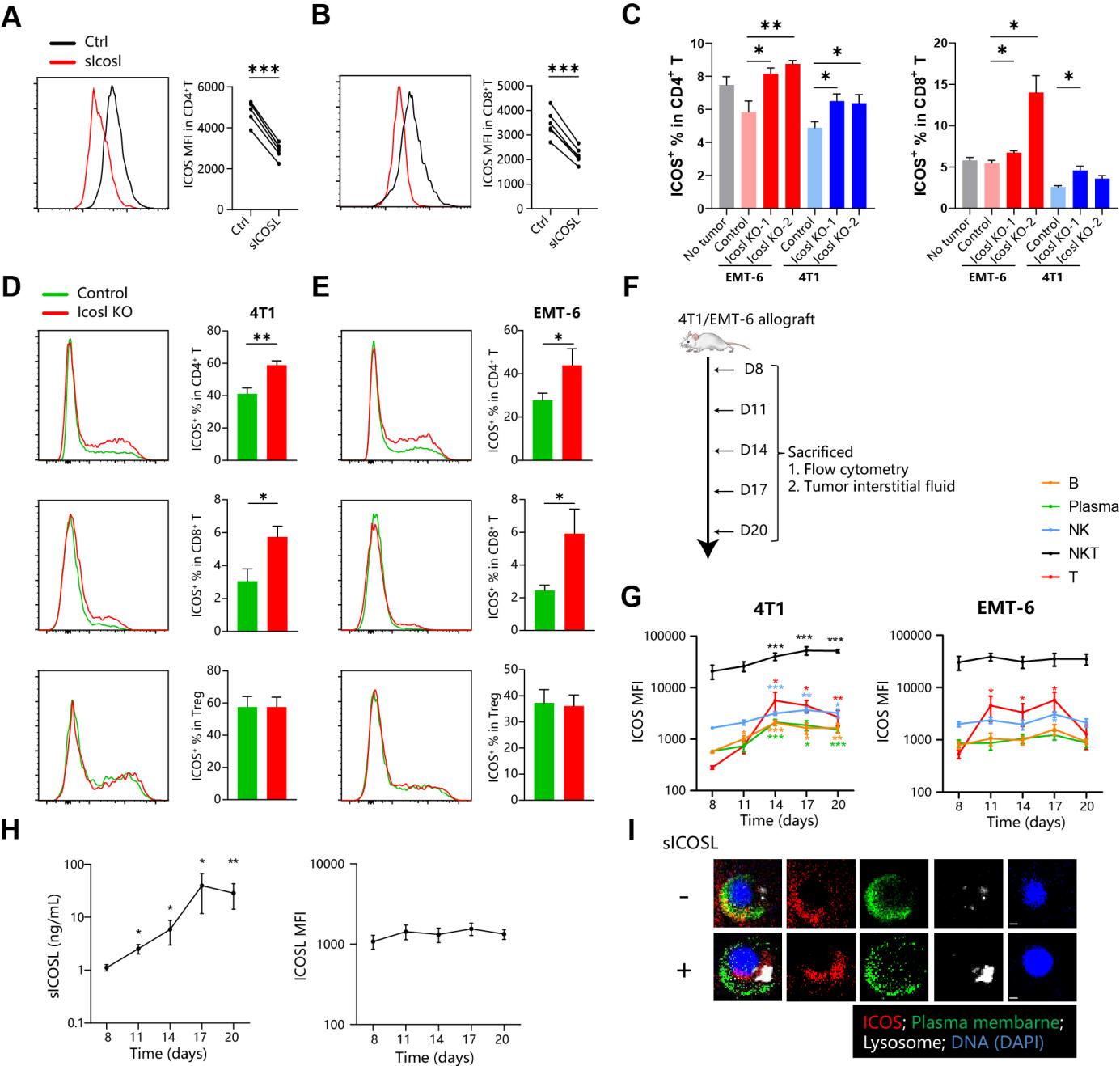


Figure S2, sICOSL promotes internalization of ICOS in T cells.

(A-B) ICOS expression of human CD4+ and CD8+ T cells after sICOSL treatment (1μg/mL) for 12 hour. (C) Icosl-KO/WT 4T1 and EMT-6 cells were cocultured with tumor-antigen activated T cells. ICOS expression in CD4+ and CD8+ T cells were evaluated. (D-E) Icosl-KO/WT 4T1 and EMT-6 tumor in immunocompetent mice were dissected. ICOS expression in different T cell subset analyzed using flow cytometry. (F-H) BALB/c mice were subcutaneously injected with 4T1 or EMT-6 cell lines, and were sacrificed in different endpoint time to dynamic monitor micorenvironment (F). ICOS expression in intratumoral lymphoid cells (G), sICOSL levels in tumor interstitial fluid and mICOSL expression in tumors (H) were detected. (I) MDA-MB-231 cells were treated with sICOSL for 0.5 h. Plasma membrane (DiI), lysosome (LysoTracker) and stained ICOS were detected by confocal microscopy. Scale bar = 2 μm.


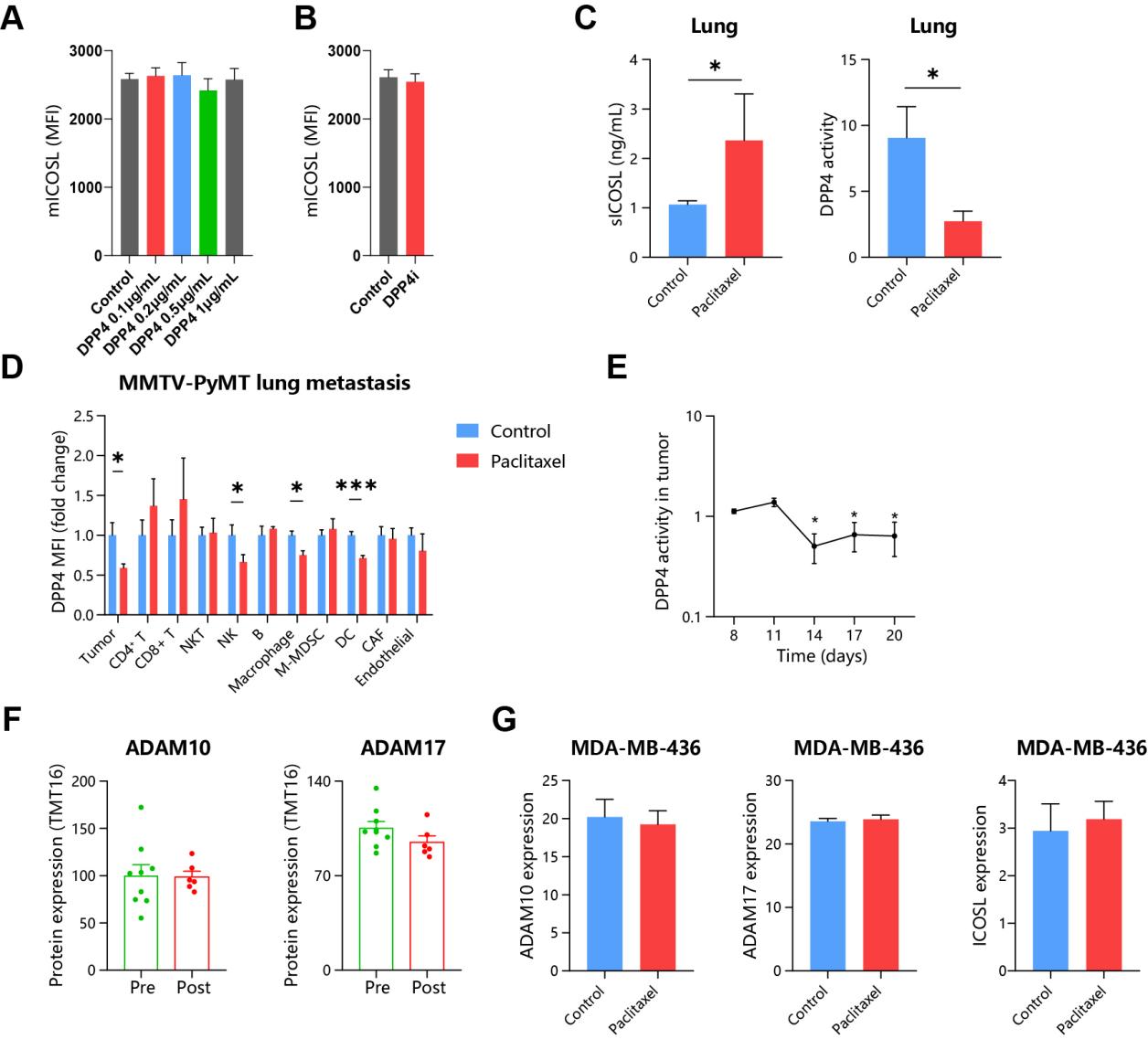


Figure S3, Chemotherapy didn’t induce sICOSL through ADAM10 and ADAM17

(A) The cancer cell was treated with varying concentrations of DPP4 for 12 hours, and the mICOSL expression was measured. (B) The cancer cell was treated with DPP4 inhibitors for 12 hours, and the mICOSL expression was measured. (C) MMTV-PyMT transgenic mice received chemotherapy. sICOSL concerntration and DPP4 activity in the lung interstitial fluid. (D) Lung were dissected and analyzed with flow cytometry. DPP4 expression in chemtherapy group were normalized based on control group for each cell cluster. (E) DPP4 activity in the tumor interstitial fluid. (F) ADAM10 and ADAM17 expression in pre- and post- chemotherapy breast cancer samples according to TMT16-based preotome. (G) ADAM10, ADAM17 and ICOSL expression in paclitaxel-treated MDA-MB-436 cells were measured through RNA-seq.


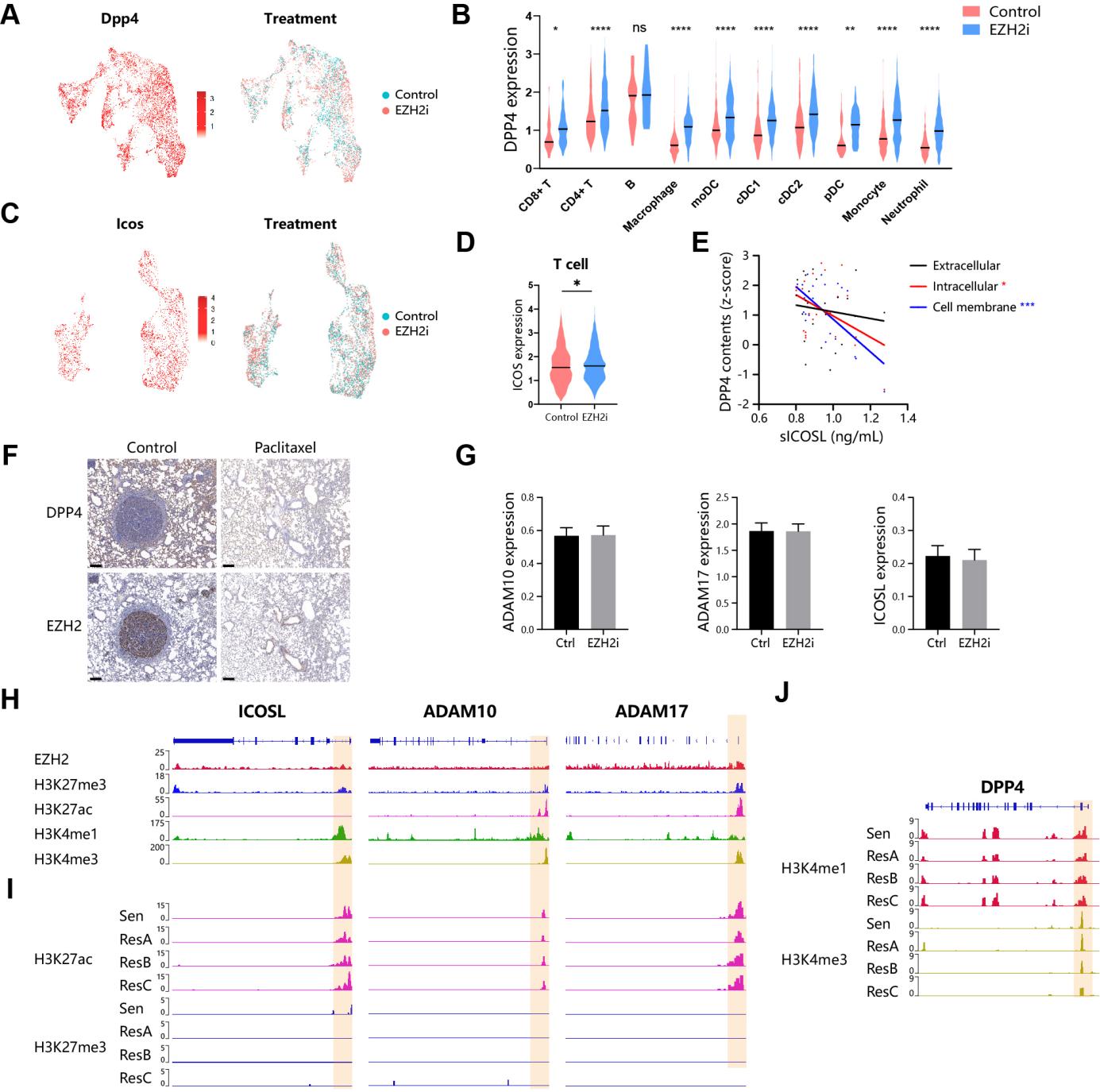


Figure S4, EZH2 mediate DPP4-sICOSL-ICOS axis

(A-D) scRNA-seq of mouse tumor-infiltrating CD45+ Cells treated with EZH2 inhibitor PF-06821497. UMAP plot of all cells which visualize Dpp4 (A) and Icos (C) expression profiles in a two-dimensional. independent space. Bar graphs show Dpp4 (B) and Icos (D) expression in different cell clusters. (E) Breast cancer cell lines were treated and detected as figure 6D-H indicated. The correlation between sICOSL concerntration with DPP4 expression in different location was reported. (F) EZH2 protein levels of lung metastasis from paclitaxel-injected MMTV-PyMT mouse. Scale bar = 100 μm. (G) ADAM10, ADAM17 and ICOSL expression of MDA-MB-436 cells after treated with EZH2i. (H) Genomic visualization of EZH2, H3K27me3, H3K27ac, H3K4me1 and H3K4me3 signal intensity of ICOSL, ADAM10 and ADAM17 gene tracks from ENCODE database. (I) Genomic visualization of H3K27ac and H3K27me3 signal intensity of ICOSL, ADAM10 and ADAM17 gene tracks in parental and paclitaxel-resistant MDA-MB-436 cells. (J) Genomic visualization of H3K4me1 and H3K4me3 signal intensity of DPP4 gene tracks in parental and paclitaxel-resistant MDA-MB-436 cells.


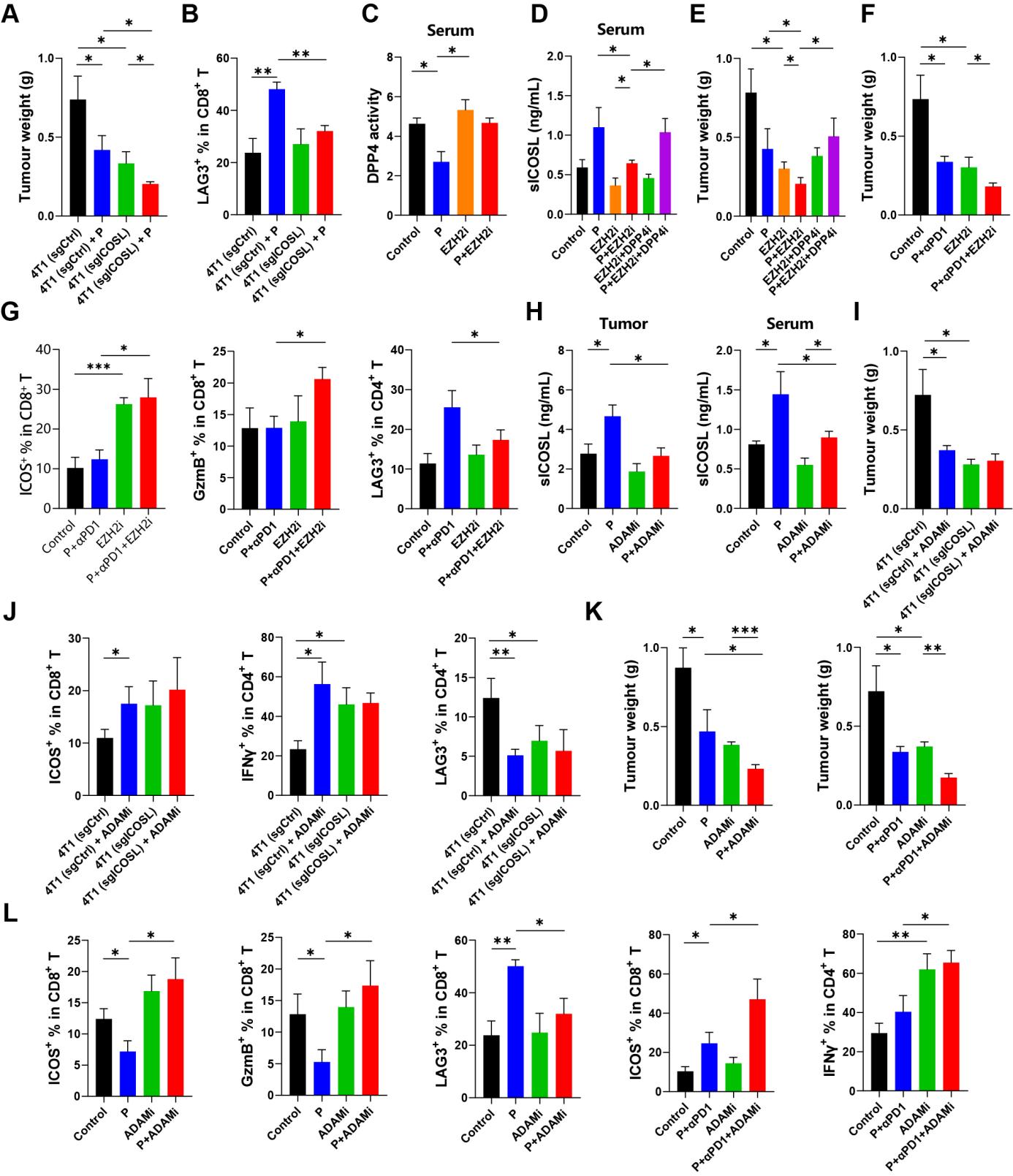


Figure S5, sICOSL blockade sensitizes the tumor to chemoimmunotherapy

(A-B) BALB/c mice inoculated with syngeneic 4T1 breast cancer cells pretransduced with sgCtrl and sgICOSL, and then were injected with paclitaxel (P), n=8 mice/group. (A) The total wet weight in grams of mammary tumors present in each mouse. (B) LAG3 positivity in CD8+ T cells. (C-G) BALB/c mice with syngeneic 4T1 breast cancer were injected with P, EZH2 inhibitor (EZH2i), DPP4 inhibitor (DPP4i) and anti-PD1 antibody (αPD1). (C) Serum DPP4 enzymatic activity. (D) Serum sICOSL concerntration. (E-F) Tumor weight. (G) ICOS, Granzyme B and LAG3 positivity in CD4+ and CD8+ T cells. (H-L) BALB/c mice with Icosl-KO or WT 4T1 breast cancer were injected with ADAMi, P and αPD1. (H) sICOSL concerntration in the serum and tumor interstitial fluid. (I, K) Tumor weight. (J, L) ICOS, IFNγ, Granzyme B and LAG3 positivity in CD4+ and CD8+ T cells.


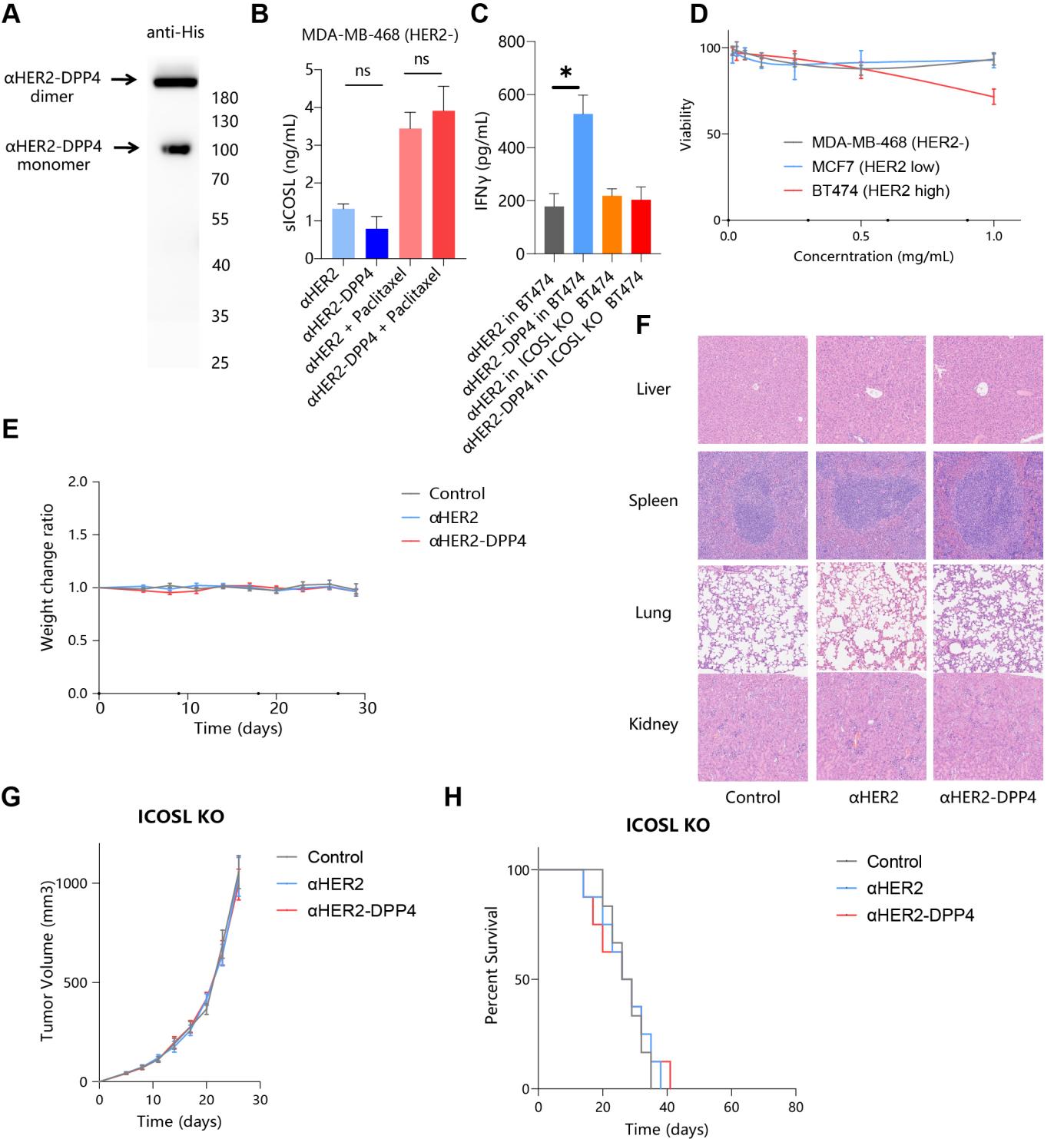


Figure S6, Characteristic and safety of nanobody-DPP4 fusions.

(A) Anti-His Western blot analysis of αHER2-DPP4 secreted from HEK293T cells. SDS-PAGE was performed under nonreducing conditions to reveal the oligomerization of the original protein. (B) sICOSL concentration in the culture supernatant of HER2-negative MDA-MB-468 cells treated with αHER2/αHER2-DPP4 and chemotherapy in vitro. (C) Tumor-antigen activated CD8+ T cells were co-cultured with control and ICOSL KO BT474 cells and treated with αHER2 or αHER2-DPP4 for 18 h. The IFNγ in the cell culture supernatant was quantified. (D) Cell viability of cell lines expressing different levels of HER2 after 24 hours of treatment with varying concentrations of αHER2-DPP4. (E-F) BALB/c mice were inoculated with syngeneic 4T1 breast cancer cells pretransduced with human HER2, and then injected with αHER2 or αHER2-DPP4. Mouse weight (E) and tissue pathology (F) are shown. (G-H) BALB/c mice were inoculated with syngeneic 4T1 breast cancer cells expressing human HER2 with ICOSL knock-out, and then injected with αHER2 or αHER2-DPP4. Tumor volume (G) and mouse survival (H) were monitored every 3 days (n = 8).


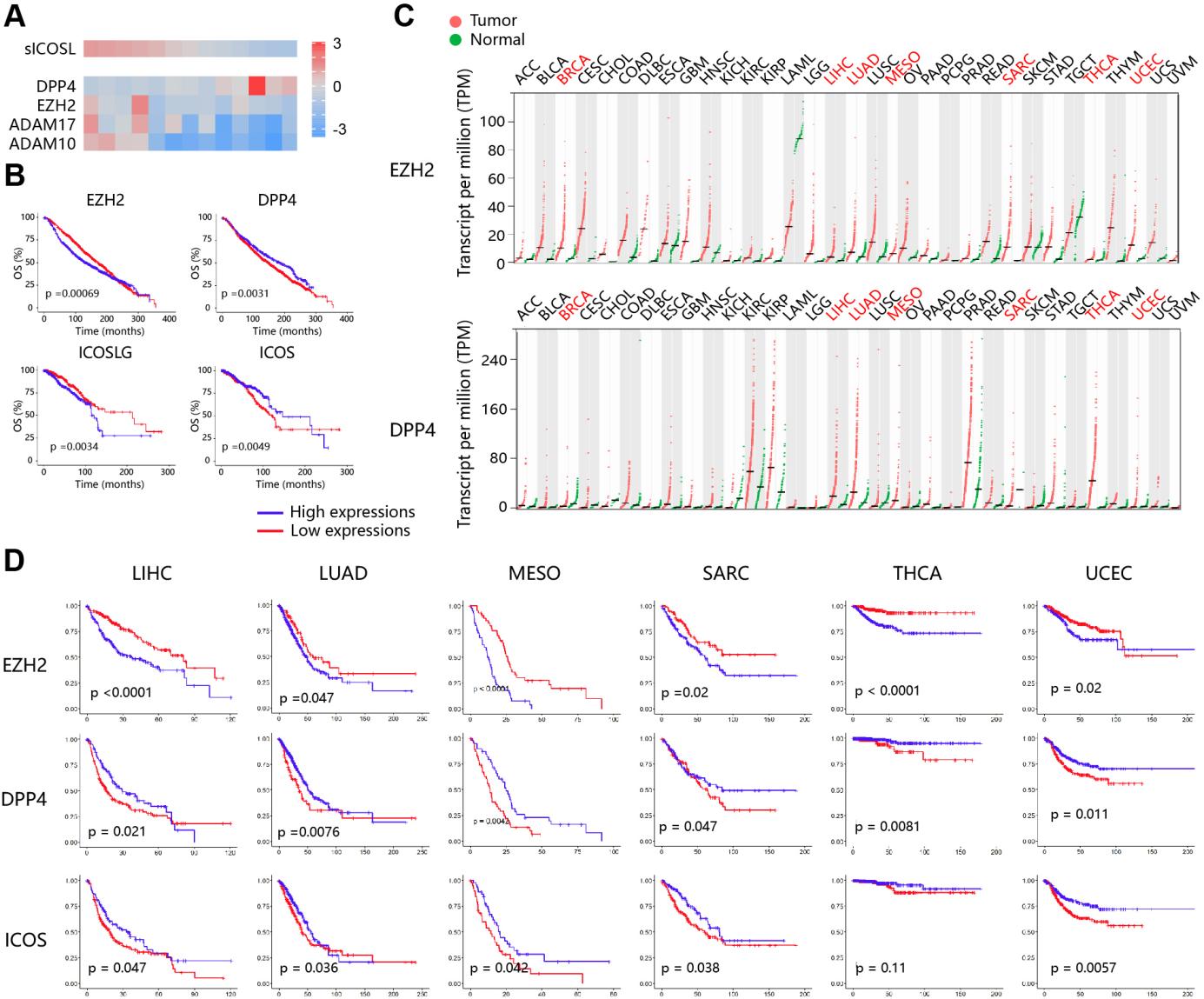


Figure S7, Pan-cancer prognosis value of sICOSL-related genes

(A) Heatmaps of Z-score of sICOSL concerntration and representative genes expression are shown. (B) Correlations of sICOSL-relevant genes with overall survival (OS) in TCGA and METABRIC. The red line designates the samples with lowly expressed genes, and the blue line indicates the samples with highly expressed genes. (C) Expression of EZH2 and DPP4 in different cancer types of TCGA cohorts. Tumors (red) and paired normal samples (green) are shown for each type. (D) Correlations of EZH2, DPP4 and ICOS in 6 cancer types with OS. The red line designates the samples with lowly expressed genes,and the blue line indicates the samples with highly expressed genes.


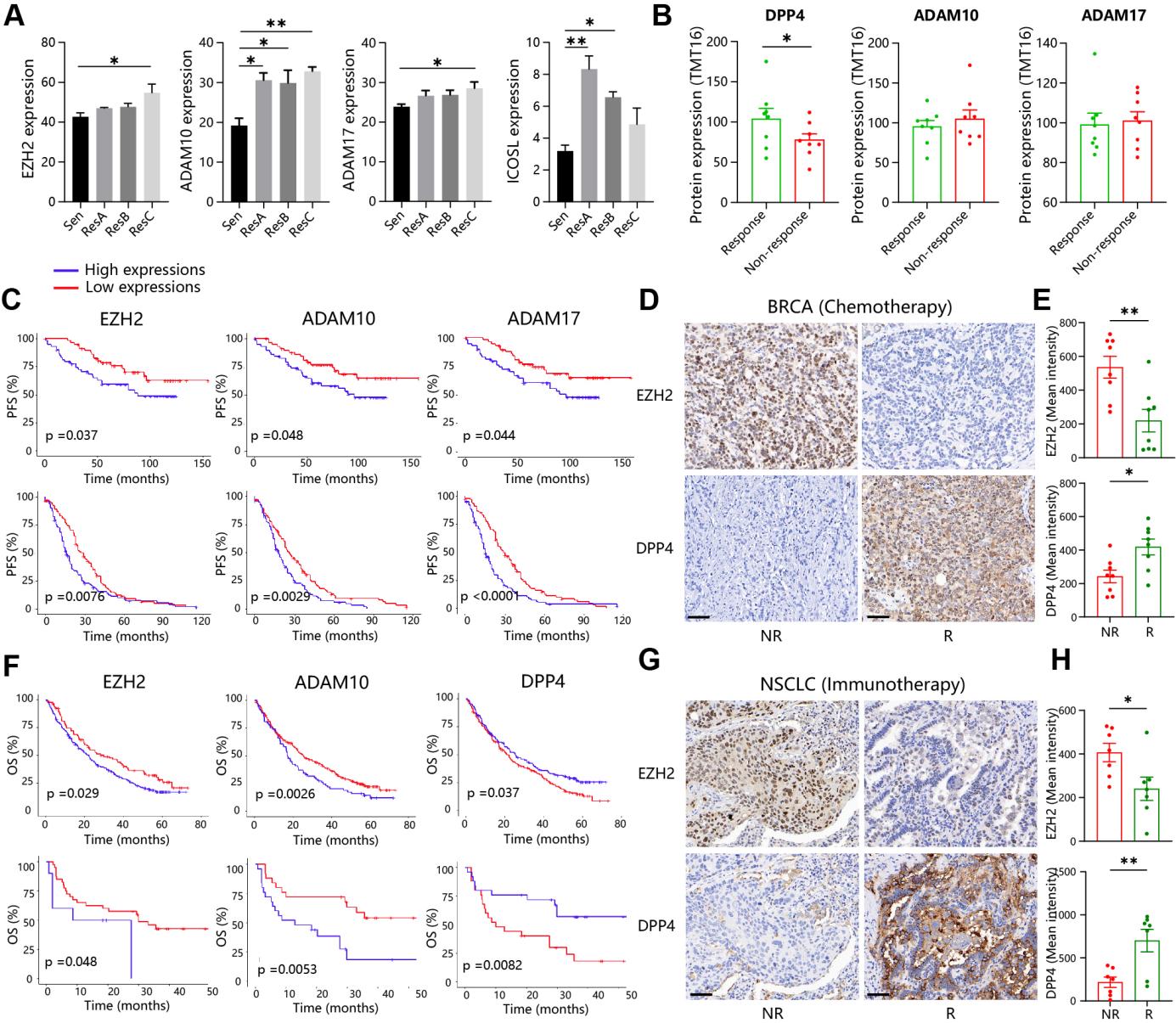


Figure S8, sICOSL-related genes in predicting chemoimmunotherapy response

(A) EZH2, ADAM10, ADAM17 and ICOSL expression in three paclitaxel-resistant (ResA/B/C) and paclitaxel-sensitive (Sen) MDA-MB-436 cell lines. (B) DPP4, ADAM10 and ADAM17 protein levels breast cancer response or not response to chemotherapy. (C) Associations of EZH2, ADAM10 and ADAM17 with PFS in two chemotherapy-treated breast cancer cohorts (Up: Loi, S., et al., Down: Bos, P.D., et al). The red line designates the samples with lowly expressed genes,and the blue line indicates the samples with highly expressed genes. (D, E) EZH2 and DPP4 protein levels of breast cancer from chemotherapy-treated patients. Scale bar = 100 μm. (F) Associations of EZH2, ADAM10 and DPP4 with OS in ccRCC (Up: Braun, D.A., et al.) and melanoma (Down: Gide, T.N., et al.) treated with immune checkpoint blockade. (G, H) EZH2 and DPP4 protein levels of NSCLC from immunotherapy-treated patients. Scale bar = 100 μm.
